# Facial width‐to‐height ratio predicts fighting success: A direct replication and extension of Zilioli et al. (2014)

**DOI:** 10.1002/ab.22027

**Published:** 2022-03-08

**Authors:** Neil R. Caton, John Hannan, Barnaby J. W. Dixson

**Affiliations:** ^1^ School of Psychology University of Queensland Brisbane Queensland Australia

**Keywords:** aggression, facial structure, facial width‐to‐height ratio, fighting ability, mixed‐martial‐arts

## Abstract

Zilioli et al. (2014) were the first to show an association between male facial width‐to‐height ratio (fWHR) and physical aggression and fighting ability in professional mixed‐martial‐arts fighters. Here, we re‐examined this relationship by replicating (using all original measures) and extending (using 23 new variables related to fighting performance) Zilioli et al. (2014) in a statistically well‐powered sample of 520 fighters using automatic and manual measures of the fWHR involving both eyelid and eyebrow landmarks, used interchangeably in previous reports (Studies 1–2). Most importantly, we successfully replicated Zilioli et al.'s (2014) central finding that fighters' fWHR, when manually calculated using the eyebrow landmark, predicted their fighting success (*p* = .004, controlling for body mass index and total fights). Consistent with past criticisms of using *fight* rather than *fighter* data to examine fighting success, which have argued that individual fights can be suddenly and unexpectedly determined and do not capture an individual's overall ability to succeed, Study 3 (*N* = 1367 fights) found no association between fWHR and singular victories. Studies 1–3 showed continual evidence that larger fWHRs were associated with grappling abilities, even after controlling for demographic and allometric factors. Strikingly, Study 3 discovered associations between all fWHR measures and grappling skill that remained robust before and after controlling for 17 different control variables. We discuss that grappling, or the act of taking down an opponent, involves a more aggressive, close‐combat approach than does striking. Combined, these results offer additional support for the argument that fWHR may have been shaped by sexual selection.

A large body of research implicates the facial width‐to‐height ratio (fWHR), measured by dividing the distance between the upper lip and brow by the distance between the left and right zygion, in a suite of behavioral outcomes (Geniole et al., 2015). While sex differences in fWHR are small (*d* = 0.11; Geniole et al., 2015), men with larger fWHRs are reportedly more antisocial (Haselhuhn & Wong, 2012), financially successful (Wong et al., 2011), aggressive (Geniole et al., 2015; Haselhuhn et al., 2015), more sexually active (Valentine et al., 2014), and have higher lifetime reproductive success (Loehr & O'Hara 2013) than men with smaller fWHRs. However, associations between facial morphology and behavioral outcomes have been disputed (e.g., Kosinski, 2017; Todorov et al., 2015; Wang et al., 2019), including whether fWHR predicts male sociosexual attributes (Dixson, 2018). This suggests a re‐evaluation of whether fWHR is an accurate indicator of male formidability is warranted.

## 1 REPLICATING ZILIOLI ET AL. (2014): FWHR AND FIGHTING SUCCESS

According to evolutionary theory, men with well‐developed masculine craniofacial morphology had greater resource‐holding potential (RHP) or the ability to win violent contests (Sell et al., 2009). Men with formidable facial structures, such as fWHR, were better able to inflict force on adversaries (Zilioli et al., 2014) which translated in greater bargaining power that could be leveraged during intra‐sexual conflict (Craig et al., 2019; Dixson et al., 2021; Sell et al., 2009, 2016). This is the leading explanation for why men with larger fWHRs show more aggression and antisocial behavior (Geniole et al., 2015; Haselhuhn et al., 2015) and is predicated on the premise that facial structure is associated with fighting success (Zilioli et al., 2014). In a proximal (but complementary) explanation, other researchers have focussed on a more proximal, underlying mechanism—hormone effects throughout development—that may lead to changes in both facial structure and key regions in the brain regulating social behavior, but this hypothesis has been controversial (Bird et al., 2016; Whitehouse et al., 2015). In the current study, then, we sought to re‐examine the hypothesis that facial structure is associated with fighting success (Zilioli et al., 2014). Hereon, we use the terms fighting success, RHP, and win percentage (i.e., total wins divided by total fights) interchangeably because win percentage is the most often used measure of fighting success and RHP throughout the human contest competition literature (e.g., Richardson & Gilman, 2019; Richardson, 2020; Třebický et al., 2013, 2019; Zilioli et al., 2014).

Testing the roles of men's secondary sexual traits in male‐male competition has benefitted from the availability of data from mixed martial arts (MMA) fighters competing in the Ultimate Fighting Championships© (UFC; Dixson et al., 2018; Pollet et al., 2013). Zilioli et al. (2014, Study 1) were the first to provide empirical evidence that MMA fighters (*N* = 241) with larger fWHRs had greater fighting success. In a subsequent commentary, Třebický et al. (2015) also reported that MMA fighters with larger fWHRs had greater fighting success using a largely overlapping database with the original study, but with a smaller sample (*N* = 146 combatants). Třebický et al. (2013) and Zilioli et al. (2014)—as well as the commentary piece on Zilioli et al. (2014) by Třebický et al. (2015)—were the first of their kind in the human contest competition literature.^1^

These studies laid the foundation for conducting research on human fighting ability from an evolutionary perspective (e.g., Aung et al., 2021; Lane & Briffa, 2020; Richardson & Gilman, 2019; Třebický et al., 2019). These studies were also the first to draw attention to the use of data from MMA fighters competing in the UFC©. This data proved to be paramount to this emerging sub‐field because contest competition research requires large sample sizes (Kasumovic et al., 2017; Richardson & Gilman, 2019) that might not be feasible when using ordinary sampling methods (e.g., simple random sampling). For example, Zilioli et al. (2014) comprised 241 UFC fighters who fought, on average, 21 professional fights. If we assume that Fighter A's win was Fighter B's loss (though professional fight data on ufc.com is not exclusively from UFC fights; see Zilioli et al., 2014, for a brief discussion), then Zilioli et al. (2014) used data aggregated from 2530.5 fights (i.e., (241 × 21)/2 = 2530.5). This is an impressive aggregation of data, which could be argued to have accurately captured fighters' RHP. While we acknowledge the possibility that the fighter's underlying number of fights on which the fighter's fight success data was based might have stabilized their estimates, fighters—rather than fights—was the unit of analysis that determined their sample size and, therefore, statistical power. Of note, positive associations between fWHR and men's behavior have indeed been criticized for relying on statistically‐underpowered sample sizes (Kosinski, 2017).

Unfortunately, Zilioli et al. (2014) and, by extension, Třebický et al. (2015) employed a sample size that was statistically‐underpowered. Table 1 presents the power analyses (conducted using G*Power 3.1; Faul et al., 2009) for Zilioli et al. (2014). Most (7 out of 9, excluding nonsignificant analyses) of the original study's analyses were below the threshold of 80% statistical power, and the average achieved power across these analyses was 0.65. Given that the power issues in Zilioli et al. (2014) and Třebický et al.'s (2015) were likely a function of the available number of UFC fighters in 2012 (when their data was collected) rather than an empirical flaw, we emphasise that Zilioli et al. (2014) and Třebický et al.'s (2015) sample sizes were expected and reasonable for their time period. However, the current study will address the limited statistical power in Zilioli et al. (2014) by sampling a larger number of UFC fighters that entered the UFC since 2012. As can be seen in Table 1, the present study's sample size was well‐positioned to perform a statistically‐powered replication of Zilioli et al. (2014).

**Table 1:** Power analyses for Zilioli et al. (2014)

Outcome variable	Zilioli et al. (2014)'s Results	Power	Required sample size for 80% power	Present study's sample size
Total number of fights	*r* = .163, *p* = .011	**0.72**	293	520
Total wins	*r* = .203, *p* = .001	0.89	188	520
Percentage of wins	*r* = .097, *p* = .132^a^	0.32^a^	831^a^	520^a^
Percentage of wins, controlling for total fights	*r* = .139, *p* = .031	**0.58**	401	520
Total fights, controlling BMI	*r* = .154, *p* = .017	**0.67**	326	520
Total wins, controlling BMI	*r* = .190, *p* = .003	0.85	212	520
Win percentage, controlling BMI	*r* = .088, *p* = .172^a^	0.28^a^	1011^a^	520^a^
Win percentage, controlling BMI and total fights	*r* = .128, *p* = .048	**0.51**	474	520
Total wins in lightweight fighters (*n* = 118)	*r* = .183, *p* = .047	**0.51**	232	265
Total wins in heavyweight fighters (*n* = 50)	*r* = .287, *p* = .043	**0.53**	93	108
Total wins in middleweight fighters (*n* = 73)	*r* = .131, *p* = .270^a^	0.20^a^	455^a^	147^a^
Win percentage in middleweight fighters (*n* = 73)	*r* = .217, *p* = .065^a^	0.46^a^	164^a^	147^a^
Win percentage in middleweight fighters, controlling total fights (*n* = 73)	*r* = .242, *p* = .040	**0.56**	129	147

*Note*: In the power column, the original study's statistically underpowered (significant) analyses have been bolded (power threshold = 0.80).

Abbreviations: BMI, body mass index; fWHR, facial width‐to‐height ratio.

^a^ It should be noted that it is not appropriate to perform post hoc power analyses on nonsignificant results because there will always be low observed‐power on nonsignificant results (Lakens, 2021); thus, we do not consider these analyses when stating that Zilioli et al.'s (2014) average observed power was 0.65. In the power column, the original study's statistically underpowered (significant) analyses have been bolded (power threshold = 0.80). Zilioli et al. (2014) also conducted analyses separately for Caucasian and non‐Caucasian fighters, but power analyses for these analyses could not be conducted because their sub‐group sample sizes were not provided in the original study. Nonetheless, these between‐ethnicity analyses were not a central focus of the present study—fWHR and fighting success, more broadly, was the central focus of the present study—and 10 out of 14 of these between‐ethnicity relations were already nonsignificant in the original study, with no clear pattern of results that showed that either Caucasian or non‐Caucasian fighters experienced greater fighting success.

With the above in mind, our first aim was to directly replicate the significant associations of Zilioli et al. (2014) using a similar, but statistically well‐powered, sample of UFC fighters. Our second broad aim was to extend the original study's findings by examining which underlying components of RHP were associated with men's fWHR. If men's fWHR is associated with fighting success most broadly, then fWHR should be associated with the components that underpin fighting success more specifically.

## 2 EXTENDING ZILIOLI ET AL. (2014): WHY DOES FWHR PREDICT FIGHTING SUCCESS?

In addition to replication, we aimed to extend the approach in Zilioli et al. (2014) through examining a larger number of variables associated with fighting ability; specifically, 23 variables new to the emerging literature on human contest competition. Zilioli et al. (2014) proposed several explanations for the association between male fWHR and fighting success, including that fWHR: (1) is associated with behavioral aggression; (2) offers blunt force protection; and (3) is associated with the ability to exert force.

### 2.1 fWHR and physical aggression

Animals most often succeed in violent fights through their ability to successfully inflict physical damage on their opponent (Sell et al., 2012). In humans, physical aggression has been defined as either the propensity to proactively or reactively inflict injuries on a conspecific potentially impeding their survival, often at a cost to the aggressor (Sell et al., 2012; Wrangham, 2018). Acts of physical aggression include inflicting damage on an opponent's oxygenating circulatory system (e.g., respiratory capacity), nervous system (e.g., damaging the organism's ability to detect, process, and respond to stimuli), and musculoskeletal system (e.g., inflicting fractures, hindering movement) (Sell et al., 2012). Fighting manoeuvres in MMA, including striking (e.g., striking accuracy, landed strikes, attempted strikes) and grappling abilities (e.g., grappling accuracy, landed takedowns, attempted takedowns, attempted submissions), could be considered as acts of aggression as they are deployed to inflict damage on the opposing respiratory, nervous, and musculoskeletal systems.

Stronger animals are more physically aggressive (Archer & Thanzami, 2007; Huntingford & Turner, 1987; Krebs & Davies, 1993; Sell et al., 2009) and individuals with larger fWHRs are more physically aggressive (Geniole et al., 2015; Noser et al., 2018). Zilioli et al. (2014) suggested that men with larger fWHRs, who are stronger fighters, should be more adept in enacting aggression because they have more bargaining power to leverage during intrasexual conflict. Logically, this should extend to the fighting domain; men with larger fWHRs experience greater fighting success because they are better to inflict damage on their opponents. Thus, we tested the association between fWHR and acts of aggression (i.e., striking/grappling abilities) during MMA contests.

### 2.2 fWHR and damage resistance

Zilioli et al. (2014) suggested that men with larger fWHRs might also experience greater fighting success because larger craniofacial structures may provide resistance to blunt force trauma. Lethal combat has been a powerful adaptive problem throughout hominin evolution, with the face being the anatomical structure most often struck and fractured during violent combat (Carrier & Morgan, 2014). Consequently, larger facial structures are one form of defensive morphology that might have evolved to solve this adaptive problem (Carrier & Morgan, 2014). Yet, no research to date has empirically examined Carrier and Morgan's (2014) protective buttressing hypothesis. Here, we examined this hypothesis by examining the relation between men's fWHR and knockout resistance (as a proxy for blunt force trauma resistance) in an extension of Zilioli et al. (2014).

### 2.3 fWHR and force output

Zilioli et al. (2014) also suggested that men's fWHR might be linked to their ability to exert force on their opponent. Physical strength—defined as the capacity to exert force to an object or opponent—might be the best predictor of fighting ability (Sell et al., 2012). Yet there is minimal theoretical reason for why fWHR should be *directly* linked to force output. Zilioli et al. (2014) suggested that developmental systems that prioritize larger (combat‐designed) bodily structures might simultaneously develop larger facial structures. There is, then, a potential allometric association that underpins the relation between fWHR and force output. MacDonell et al. (2018) demonstrated that men with larger fWHRs are physically stronger (measured as greater bicep circumference) and men with greater bicep circumference can exert greater force (Smith et al., 2008). Indirectly then, men's fWHR may be associated with their force output (Zilioli et al., 2014).

Men's fWHR may not *directly* predict their force output (especially as UFC limits fighters to weight categories) but could be associated with their underlying anatomical components that collectively contribute to force output. Thus, our study employed two methods for examining the relation between fWHR and potential force output: (1) by directly examining the association between fWHR and knockout wins (a proxy for force output) and (2) by examining the association between fWHR and morphological structures implicated in force output (e.g., overall body size, also called weight). Previous research has indeed interpreted links between physiological features (e.g., vocal parameters) and bodily size as evidence for the physiological feature being an indicator of the individual's RHP (Aung et al., 2021).

## 3 THE PRESENT WORK

In the present work, we conducted three studies that aimed to directly replicate and extend the findings of Zilioli et al. (2014). In Study 1, we aimed to replicate the significant associations of Zilioli et al. (2014) using a statistically well‐powered sample of 520 UFC fighters using computer‐automated fWHR measurements. Recent research suggests an advantage, when using large datasets, in employing anthropometric measurements generated automatically using programmed algorithms (Jones et al., 2021). Study 1 also extended Zilioli et al. (2014) by examining whether a suite underlying components associated RHP were also positively associated with men's fWHR: physical aggression, blunt‐force trauma resistance, and/or force output.

Similar to Study 1, Study 2 also aimed to directly replicate and extend Zilioli et al. (2014). Study 2 only differed to Study 1 in that Study 2 used manual fWHR measurements. While automatic measures are strongly associated with manual calculations (*r* = .86: Kosinski, 2017; *r* = .91: Schild et al., 2019), Kosinski (2017) reported some variation between manual and automatic measurements (e.g., variation between females fWHR and self‐reported extraversion in manual, *p* = .032, but not in automatic, *p* = .052, measures). Further, de Kok (2017) suggested that the fWHR calculator might be subject to slight misalignment.

Study 3 aimed to address the debatable violation of independence of observations in overall fighter data (e.g., Fighter A's win might be Fighter B's loss). Study 3 extended Zilioli et al. (2014) by examining the associations between fWHR and fighting success, aggression, blunt‐force trauma resistance, and force output, using contest data among individual fighters—the largest individual fight dataset to date (see Dixson et al., 2018; Lane & Briffa, 2020). In so doing, our third study served as: (1) a conceptual replication of Zilioli et al. (2014) for the links between fWHR and fighting success; and (2) an extension, in that we further sought to examine the links between fWHR and aggression, blunt‐force resistance, and force output.

## 4 STUDY 1: AUTOMATIC FWHR MEASUREMENTS

### 4.1 Method: Study 1

#### 4.1.1 Participants and procedure

Data were gathered on all 734 UFC MMA fighters from ufc.com or espn.com up to April 4th, 2020. In line with the original study, we excluded fighters without facial photographs (*n* = 19) and female fighters (*n* = 113). An independent research assistant then coded these facial photographs in accordance with Zilioli et al.'s (2014) exclusion criteria. Thus, we excluded fighters based on non‐neutral facial expressions (*n* = 5), hair/beards that covered the zygions (*n* = 15), head tilt (*n* = 17), and UFC retirement/termination (*n* = 45), leaving a final sample of 520 fighters. While our data analyses hereafter employs this sample of 520 fighters, we ran additional analyses with the retired/terminated fighters (see ESM). In line with Zilioli et al. (2014), which followed on from Třebický et al. (2013), an independent research assistant also coded whether fighters were perceived as Caucasian (*n* = 353) or non‐Caucasian (*n* = 167). Zilioli et al. (2014) conducted additional analyses (split by perceived ethnicity) for greater comprehensiveness, in light of Třebický et al.'s (2013) finding that fighters' craniofacial morphology (using geometric morphometrics) predicted fighting success in Caucasian fighters.

In line with the original study, we collected data on fighters' wins (*M* = 15.02; *SD* = 6.56), total fights (*M* = 19.52; *SD* = 9.32), weight (*M* lbs = 169.59; *SD* = 35.32), and height (*M* inches = 70.64; *SD* = 3.33). To directly replicate the original study, which controlled for body mass index (BMI) rather than weight or height themselves, we calculated BMI (*M* = 23.70; *SD* = 3.42) using the formula: weight (lbs)/(height[inches] × height[inches]) × 703 (with 703 being the number used to convert lbs/inches^2^ to kg/m^2^). In accord with the original study, we calculated fight success (*M* = 0.79; *SD* = 0.11) using previously published methods (Třebický et al., 2013, 2019; Zilioli et al., 2014) whereby the total number of wins were divided by the total number of fights.^2^

Two research assistants then coded fighters' fWHR from their facial photographs using the automatic fWHR calculator in Python (de Kok, 2017) for both eyebrow (*M* = 1.73; *SD* = 0.15) and eyelid (*M* = 1.94; *SD* = 0.17) fWHR measurements. There is no variation between research assistants in this process because the fWHR code is simply entered into the terminal and the fWHR calculator consequently provides the resulting fWHR statistic. Both measurements were strongly associated (*r* = .85). The fWHR calculator extracts the bizygomatic and eyebrow/eyelid landmarks that have been placed by the face recognition package, and divides the individual's bizygomatic width by their facial height (eyelid or eyebrow, depending on which is specified). Given that Zilioli et al. (2014) only ran statistics for fWHRbrow, our results section below focussed on the fWHRbrow analyses. Our study also extended the original study by examining fWHRlid, which demonstrated weaker results (see ESM).

#### 4.1.2 Measures

##### Demographic and physical measures

We collected data on fighters' age (in years; *M* = 30.27; *SD* = 4.19), arm reach (in inches; *M* = 72.73; *SD* = 4.04), leg reach (in inches; *M* = 40.54; *SD* = 3.04), and debut date (with debut dates ranging from 15th May, 1998, to 9th November, 2019). Zilioli et al. (2014) did not collect these measures, and so we did not include these measures when directly replicating the original study's analyses. However, these variables were included (either as covariates or outcome variables) when extending our analyses to examine aggression, blunt force trauma resistance, and force output.

##### Aggression, blunt force trauma resistance, and force output

We collected all available data on ufc.com and espn.com for proxies of aggression, blunt force trauma resistance, and force output. Blunt force trauma resistance was measured via fighters' cumulative number of losses by knockout/technical knockout (KO/TKO) whereas force output was measured via fighters' cumulative wins by KO/TKO.

Additionally, we collected data on 15 measures of striking (i.e., striking accuracy; significant strikes landed; significant strikes attempted; significant strikes landed per minute; significant strikes landed in a standing position, clinch position, and ground position; significant strikes landed to the opponent's head, body, and legs) and grappling abilities (i.e., grappling accuracy; takedowns landed; takedowns attempted; takedowns landed per 15 min; submission attempts per 15 min), which allowed us to extend the original study by examining the relations between fWHR and acts of aggression.

We also included multiple exploratory variables available from ufc.com and espn.com, including striking defense, takedown defense, wins and losses by submission, and wins and losses by decision. Definitions taken from James et al. (2017) and Kirk (2018) and descriptive statistics for these variables are included in the ESM. Data for Studies 1 and 2 are available on the Open Science Framework (OSF; https://osf.io/scde7/).

### 4.2 Results: Study 1

#### 4.2.1 Direct replication of Zilioli et al. (2014)

##### fWHR eyebrow

All analyses followed Zilioli et al. (2014) for the fWHRbrow (eyebrow) measurement (see ESM for identical analyses for fWHRlid). There were no statistically significant associations between fWHR and fighting success indicators (Table 2). However, associations between win percentage and fWHRbrow, controlling for the total number of fights, and then controlling for both BMI and the total number of fights, approached conventional statistical significance. Following Zilioli et al. (2014), we also ran correlations between fWHRbrow and fighting success within weight categories. The weight categories and sample sizes were as follows; in lightweight (from 57 to 70 kg, *n* = 265), middleweight (from 77 to 84 kg, *n* = 147), and heavyweight (from 90 kg to 120 kg, *n* = 108) fighters. There were no statistically significant relationships when restricting analyses to weight categories (Table 2).

**Table 2:** Correlations between fWHRbrow and fighting performance

Analysis	Original study		Present study	
	*r*	*p*	*r*	*p*
Total fights	.16	.01	.04	.40
Total wins	.20	.001	.05	.31
Win percentage	.10	.13	.06	.17
Win percentage, controlling for total fights	.14	.03	.08	.06
Total fights, controlling for BMI	.15	.02	.05	.23
Total wins, controlling for BMI	.19	.003	.06	.17
Win percentage, controlling for BMI	.09	.17	.05	.22
Win percentage, controlling for BMI and total fights	.13	.048	.08	.06
Split by weight category				
Total fights (lightweight)	N/A^a^	N/A^a^	.04	.48
Total fights (middleweight)	N/A^a^	N/A^a^	.04	.59
Total fights (heavyweight)	N/A^a^	N/A^a^	.05	.59
Total wins (lightweight)	.18	.047	.05	.38
Total wins (middleweight)	.13	.27	.04	.62
Total wins (heavyweight)	.29	.04	.07	.50
Win percentage (lightweight)	N/A^a^	N/A^a^	.06	.37
Win percentage (middleweight)	.22	.07	.01	.88
Win percentage (heavyweight)	N/A^a^	N/A^a^	.09	.33
Win percentage, controlling total fights (lightweight)	N/A^a^	N/A^a^	.08	.19
Win percentage, controlling total fights (middleweight)	.24	.04	.04	.65
Win percentage, controlling total fights (heavyweight)	N/A^a^	N/A^a^	.12	.24

*Note*: Statistically significant values have been bolded.

Abbreviations: BMI, body mass index; fWHR, facial width‐to‐height ratio.

^a^ These analyses were not performed in Zilioli et al. (2014) but we have conducted these analyses for complete clarity to the reader.

We then restricted analyses to within ethnicities (Caucasian, non‐Caucasian) and then among fighters of different ethnicities within each weight category (Table 3). All associations were nonsignificant. For comprehensiveness, we ran all the same analyses including the retired/terminated fighters for fWHRbrow (ESM).

**Table 3:** Correlations between fWHRbrow and fighting performance, split by ethnicity

Analysis	Original study		Present study	
	*r*	*p*	*r*	*p*
Caucasian fighters				
Total wins	.13	.13	.01	.81
Total fights	.06	.51	.02	.73
Win percentage	.21	.02	.02	.72
Win percentage, controlling for total fights	.23	.01	.03	.58
Total fights (lightweight)	N/A^a^	N/A^a^	.02	.83
Total fights (middleweight)	N/A^a^	N/A^a^	−.04	.71
Total fights (heavyweight)	N/A^a^	N/A^a^	.12	.33
Total wins (lightweight)	.15	.25	.00	.98
Total wins (middleweight)	.01	.96	−.04	.65
Total wins (heavyweight)	.27	.20	.13	.30
Win percentage (lightweight)	N/A^a^	N/A^a^	.02	.75
Win percentage (middleweight)	N/A^a^	N/A^a^	−.02	.87
Win percentage (heavyweight)	N/A^a^	N/A^a^	.06	.62
Win percentage, controlling total fights (lightweight)	N/A^a^	N/A^a^	.04	.64
Win percentage, controlling total fights (middleweight)	N/A^a^	N/A^a^	−.04	.64
Win percentage, controlling total fights (heavyweight)	N/A^a^	N/A^a^	.09	.48
Non‐Caucasian fighters				
Total wins	.28	.003	.09	.23
Total fights	.28	.003	.08	.33
Win percentage	.02	.86	.10	.19
Win percentage, controlling for total fights	.02	.81	.14	.08
Total fights (lightweight)	N/A^a^	N/A^a^	.11	.30
Total fights (middleweight)	N/A^a^	N/A^a^	.31	.06
Total fights (heavyweight)	N/A^b^	N/A^a^	−.06	.74
Total wins (lightweight)	N/A^b^	N/A^b^	.14	.17
Total wins (middleweight)	N/A^b^	N/A^b^	.31	.06
Total wins (heavyweight)	N/A^b^	N/A^b^	−.05	.78
Win percentage (lightweight)	N/A^a^	N/A^a^	.06	.60
Win percentage (middleweight)	N/A^a^	N/A^a^	.07	.65
Win percentage (heavyweight)	N/A^a^	N/A^a^	.16	.34
Win percentage, controlling total fights (lightweight)	N/A^a^	N/A^a^	.09	.38
Win percentage, controlling total fights (middleweight)	N/A^a^	N/A^a^	.19	.26
Win percentage, controlling total fights (heavyweight)	N/A^a^	N/A^a^	.15	.38

Abbreviation: fWHR, facial width‐to‐height ratio.

^a^ These analyses were not performed in Zilioli et al. (2014) but we have conducted these analyses for complete clarity to the reader.

^b^ For the associations between total wins across weight categories for non‐Caucasian fighters, Zilioli et al. (2014) did not provide specific statistical details for each analysis but broadly note that, “These correlations were also not significant among non‐Caucasian fighters (average *r* = .27, lowest *p* = .090)” (p. 325).

#### 4.2.2 Exploratory analyses: Career stage and fWHR

Thus far, we report limited evidence for associations between fWHR and formidability. However, fighters might adjust their strategies in response to their opponent. For example, fighters with large fWHRs with prior wins by knockout earlier in their career, subsequent opponents might strategically avoid stand‐up combat, potentially suppressing any associations between fWHR and fighting success. If the relationship between fWHR and fighting success is stronger among early than late‐career fighters, then fighters' debut date may moderate the relationship between fWHR and fighting success. Using Hayes' (2013) SPSS PROCESS macro (model 1; v.3.5; 10000 bootstrap samples; 95% bias‐corrected confidence intervals), four moderation analyses were performed to examine the effect of debut date on the relationship between fWHRbrow and a suite of outcome variables (total fights, total wins, win percentage, win percentage controlling for total fights). Debut date did not significantly moderate the relationship between fWHRbrow and fight success (total fights: standardised interaction *B* = −0.04, *SE* = 0.03, *t* = −1.23, *p* = .22; total wins: standardised interaction *B* = −0.03, *SE* = 0.03, *t* = −1.03, *p* = .30; win percentage: standardised interaction *B* = 0.02, *SE* = 0.04, *t* = 0.45, *p* = .65; win percentage, controlling for total fights: standardised interaction *B* = −0.03, *SE* = 0.05, *t* = 0.16, *p* = .87).

#### 4.2.3 Extension of Zilioli et al. (2014)

Statistical associations between fWHR and the 23 measures of fighting abilities—as proxies for aggression, blunt force trauma resistance, and force output, as well as the exploratory variables—are reported in Table 4. There was a significant association between time‐adjusted landed takedowns before and after controlling for the covariates. This same pattern of results emerged when we included the retired fighters (see ESM). For interested readers, a correlation matrix including fWHR, win percentage, and all the covariates is included in the ESM. Of note, there was a significant, positive association between fWHR and weight.

**Table 4:** Correlations between automatic fWHR and fighting abilities

	fWHR (eyelid)				fWHR (eyebrow)			
Outcome variable	Bivariate^a^		Partial^b^		Bivariate^a^		Partial^b^	
	*r*	*p*	*r*	*p*	*r*	*p*	*r*	*p*
Striking abilities								
Striking accuracy	.05	.29	.02	.67	.05	.32	−.00	.97
Total strikes landed	.01	.86	.04	.42	.02	.66	.01	.80
Total strikes attempted	−.01	.87	.03	.61	.01	.85	.01	.93
Strikes landed per minute	.01	.79	.00	.99	.00	.93	−.00	.98
Strikes landed in standing position	.01	.81	.05	.31	.01	.76	.02	.77
Strikes landed in clinch position	.00	.99	.01	.88	.03	.58	.01	.90
Strikes landed in ground position	.00	.94	.00	.95	.02	.74	−.00	.95
Strikes landed to the opponent's head	.01	.85	.04	.45	.02	.71	.00	.94
Strikes landed to the opponent's body	.00	.96	.04	.48	.02	.65	.02	.65
Strikes landed to the opponent's legs	.00	.99	.03	.60	.00	.93	.00	.97
Grappling abilities								
Grappling accuracy	.04	.44	.05	.39	.04	.46	.04	.42
Total takedowns landed	.04	.39	.07	.21	.07	.18	.08	.14
Total takedowns attempted	.01	.83	.02	.65	.03	.53	.03	.62
Landed takedowns per 15 min	.06	.20	.05	.34	**.13**	**.01**	**.12**	**.02**
Attempted submissions per 15 min	.02	.73	−.01	.93	.08	.14	.07	.25
Defensive abilities								
Striking defense	−.03	.52	.02	.70	−.03	.59	.02	.65
Takedown defense	−.06	.22	−.09	.10	−.03	.57	−.05	.38
Losses by KO/TKO	−.01	.83	−.03	.61	.02	.67	−.03	.51
Losses by submission	−.04	.39	−.04	.48	−.00	.93	−.03	.61
Losses by decision	.00	.93	.02	.67	.01	.90	−.01	.82
Fighting wins by type								
Wins by KO/TKO	.04	.40	.00	.95	.01	.79	−.05	.29
Wins by submission	−.06	.23	−.01	.86	.06	.18	.07	.15
Wins by decision	−.01	.77	.03	.57	.02	.72	.03	.53

Abbreviations: fWHR, facial width‐to‐height ratio; KO/TKO, knockout/technical knockout; UFC, Ultimate Fighting Championships.

^a^ Bivariate column represents the bivariate correlations between fWHR and each outcome variable.

^b^ Partial column represents the partial correlations between fWHR and each outcome variable, with age, reach, leg reach, debut date, total fights, weight, and height partialled out. Pairwise deletion was used. We considered it to be theoretically important to control for both age and debut date, as fighters can enter the UFC at a later age because they might come out of another professional organisation (e.g., NBA, NFL, WWE).

### 4.3 Discussion: Study 1

There were no significant relations between fWHR and fighting success. These results support other studies reporting a lack of association between fWHR and men's behavior (Kosinski, 2017), while contrasting with previous research among professional MMA fighters (Zilioli et al., 2014). Of note, men with larger fWHRs deployed more (time‐adjusted) landed takedowns, which remained robust when controlling for demographic and allometric factors, providing some evidence for an association between fWHR and aggression. We found no evidence an association between fWHR and blunt‐force trauma resistance. While we also failed to find support for a direct link between fWHR and force output (knockout power), there was a significant relationship between fWHR and weight, which may indicate force output (Sell et al., 2012, 2016).

**Figure 1:**
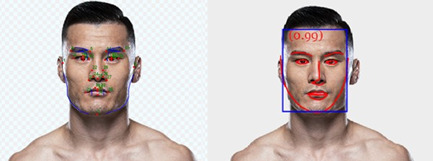
Facial landmarking for manual (tpsDig2) and automatic (facial width‐to‐height ratio [fWHR] calculator) measurements note. fWHR calculator image was taken from https://www.tiesdekok.com/calculatefwhr/[Color figure can be viewed at wileyonlinelibrary.com]

While the previous study was almost identical to the original study, we used computer‐automated fWHR measurements whereas Zilioli et al. (2014) used a manual (eyebrow) fWHR measurement. While automatic measures are strongly associated with manual calculations (*r* = .86: Kosinski, 2017; *r* = .91: Schild et al., 2019), there is some variation between females fWHR and self‐reported extraversion in manual (*p* = .032) but not in automatic (*p* = .052) measures (Kosinski, 2017). Although there were no significant associations between fWHR and fighting success, relationships between fWHRbrow and win percentage (1: controlling for total fights; 2: controlling for total fights and BMI) were approaching conventional statistical significance. Thus, our unsuccessful replication of Zilioli et al. (2014) might be due to our use of automatic measures, where manual measurements were used in the original study. Indeed, de Kok (2017) suggested that the fWHR calculator might be subject to slight misalignment (see Figure 1 below). For this reason, Study 2 sought to replicate Study 1 using manual fWHR measurements.

## 5 STUDY 2: MANUAL FWHR MEASUREMENTS

### 5.1 Method: Study 2

The methods were identical to Study 1, except for the use of manual landmarking procedures. After the completion of Study 1, we had also finished collecting landmarking data for a larger project (Caton et al., 2021) which was a preregistered direct replication of Třebický et al. (2013). This project aimed to examine the links between facial structure (using geometric morphometrics), fighting success, perceived aggressiveness, and perceived fighting ability (Caton et al., 2021). In this larger project, seven independent research assistants performed facial landmarking procedures on the entire original dataset of 715 faces (i.e., 102 faces per research assistant) in the tpsDig2 software (version 2.31; Rohlf, 2018) using 35 landmarks (i.e., anatomically homologous points that represent similar positions in separate stimuli) and 36 semi‐landmarks (i.e., curves and outlines situated between two separate landmarks). The positions of these landmarks and semi‐landmarks were informed by Třebický et al. (2013) and, therefore, the Třebický et al. (2015) commentary piece on Zilioli et al. (2014). Figure 1 shows the order of landmarking employed and compares them to the manual facial landmarking performed in tpsDig2 to the output of the de Kok's (2017) fWHR calculator. This shows that the fWHR calculator can be subject to misalignment, even for well‐aligned faces.

In line with de Kok's (2017) calculator, we calculated: (1) bizygomatic width as the 3rd landmark's x‐coordinate subtracted from the 1st landmark's x‐coordinate (i.e., the third landmark x‐coordinate minus the first landmark's x‐coordinate; we also considered the underlying semi‐landmarks' x‐coordinates, although this did not change the significance of the results, possibly as they are situated on very similar positions on the x‐axis); (2) facial height (eyebrow) as the average y‐coordinate of the semi‐landmarks (semi‐landmarks function as landmarks unless otherwise specified) to the upper‐right and left of the 4th and 13th landmarks, respectively, subtracted from the average y‐coordinate of the 30th and 32nd landmarks; (3) facial height (eyelid) as the average y‐coordinate of the 8th and 17th landmarks subtracted from the average y‐coordinate of the 30th and 32nd landmarks. Bizygomatic width was then divided by facial height (eyebrow) to create fWHRbrow, and divided by facial height (eyelid) to create fWHRlid. As with the automatic measurements in Study 1, both the manual fWHRbrow and fWHRlid measurements were strongly correlated (*r* = .78, *p* < .001). The manual fWHRbrow measurement was also strongly correlated with the automatic fWHRbrow measurement (*r* = .65, *p* < .001), as was the manual fWHRlid measurement with the automatic fWHRlid measurement (*r* = .75, *p* < .001). For the direct replication aspect, fWHRlid associations are reported in the ESM because Zilioli et al. (2014) did not use fWHRlid measurements. Manual landmarking data and facial stimuli for the larger project are publicly available on the OSF (https://osf.io/5v6mn/).

### 5.2 Results: Study 2

#### 5.2.1 Direct replication of Zilioli et al. (2014)

##### fWHR eyebrow

Table 5 presents the associations between manual fWHRbrow measure and fighting success indicators. There were significant associations between fWHRbrow and win percentage, win percentage controlling for total fights, win percentage controlling for BMI, and win percentage controlling for total fights and BMI. There was also an association between fWHRbrow and win percentage controlling for total fights in middleweight fighters. All other associations were nonsignificant.

**Table 5:** Correlations between fWHRbrow and fighting success indicators

Analysis	Original study		Present study	
	*r*	*p*	*r*	*p*
Total fights	**.16**	**.01**	−.03	.54
Total wins	**.20**	**.001**	−.00	.99
Win percentage	.10	.13	**.12**	**.005**
Win percentage, controlling for total fights	**.14**	**.03**	**.12**	**.005**
Total fights, controlling for BMI	**.15**	**.02**	‐.01	.90
Total wins, controlling for BMI	**.19**	**.003**	.02	.62
Win percentage, controlling for BMI	.09	.17	**.12**	**.008**
Win percentage, controlling for BMI and total fights	**.13**	**.048**	**.13**	**.004**
Split by weight category				
Total fights (lightweight)	N/A^a^	N/A^a^	−.03	.65
Total fights (middleweight)	N/A^a^	N/A^a^	.11	.18
Total fights (heavyweight)	N/A^a^	N/A^a^	−.01	.89
Total wins (lightweight)	**.18**	**.047**	−.01	.87
Total wins (middleweight)	.13	.27	.15	.06
Total wins (heavyweight)	**.29**	**.04**	.02	.85
Win percentage (lightweight)	N/A^a^	N/A^a^	.10	.11
Win percentage (middleweight)	.22	.07	.11	.20
Win percentage (heavyweight)	N/A^a^	N/A^a^	.14	.14
Win percentage, controlling total fights (lightweight)	N/A^a^	N/A^a^	.12	.10
Win percentage, controlling total fights (middleweight)	**.24**	**.04**	**.18**	**.03**
Win percentage, controlling total fights (heavyweight)	N/A^a^	N/A^a^	.14	.14

*Note*: Statistically significant values have been bolded.

Abbreviations: BMI, body mass index; fWHR, facial width‐to‐height ratio.

^a^ These analyses were not performed in Zilioli et al. (2014) but we have conducted these analyses for complete clarity to the reader.

We then restricted analyses to within ethnicities (Caucasian, non‐Caucasian) and then among fighters of different ethnicities within each weight category (Table 6). There were significant associations between fWHRbrow and win percentage in non‐Caucasian fighters, and win percentage controlling for total fights in Caucasian fighters. There was also a significant association between win percentage, controlling for total fights, in middleweight Caucasian fighters. All other associations were nonsignificant. For comprehensiveness, we ran all the same analyses including the retired/terminated fighters for fWHRbrow (ESM).

**Table 6:** Correlations between fWHRbrow and fighting success indicators across ethnicity

Analysis	Original study		Present study	
	*r*	*p*	*r*	*p*
Caucasian fighters				
Total wins	.13	.13	.03	.56
Total fights	.06	.51	.00	.99
Win percentage	**.21**	**.02**	.10	.06
Win percentage, controlling for total fights	**.23**	**.01**	**.11**	**.04**
Total fights (lightweight)	N/A^a^	N/A^a^	−.02	.79
Total fights (middleweight)	N/A^a^	N/A^a^	.10	.29
Total fights (heavyweight)	N/A^a^	N/A^a^	.04	.77
Total wins (lightweight)	.15	.25	−.02	.77
Total wins (middleweight)	.01	.96	.16	.10
Total wins (heavyweight)	.27	.20	.08	.49
Win percentage (lightweight)	N/A^a^	N/A^a^	.06	.47
Win percentage (middleweight)	N/A^a^	N/A^a^	.12	.24
Win percentage (heavyweight)	N/A^a^	N/A^a^	.14	.26
Win percentage, controlling total fights (lightweight)	N/A^a^	N/A^a^	.06	.47
Win percentage, controlling total fights (middleweight)	N/A^a^	N/A^a^	**.20**	**.04**
Win percentage, controlling total fights (heavyweight)	N/A^a^	N/A^a^	.15	.23
Non‐Caucasian fighters				
Total wins	**.28**	**.003**	−.07	.39
Total fights	**.28**	**.003**	−.08	.29
Win percentage	.02	.86	**.16**	**.04**
Win percentage, controlling for total fights	.02	.81	.14	.07
Total fights (lightweight)	N/A^a^	N/A^a^	−.03	.78
Total fights (middleweight)	N/A^a^	N/A^a^	.18	.29
Total fights (heavyweight)	N/A^a^	N/A^a^	−.10	.54
Total wins (lightweight)	N/A^b^	N/A^b^	.01	.91
Total wins (middleweight)	N/A^b^	N/A^b^	.16	.33
Total wins (heavyweight)	N/A^b^	N/A^b^	−.10	.55
Win percentage (lightweight)	N/A^a^	N/A^a^	.15	.17
Win percentage (middleweight)	N/A^a^	N/A^a^	.07	.68
Win percentage (heavyweight)	N/A^a^	N/A^a^	.16	.35
Win percentage, controlling total fights (lightweight)	N/A^a^	N/A^a^	.14	.18
Win percentage, controlling total fights (middleweight)	N/A^a^	N/A^a^	.13	.43
Win percentage, controlling total fights (heavyweight)	N/A^a^	N/A^a^	.13	.46

*Note*: Statistically significant values have been bolded.

Abbreviation: fWHR, facial width‐to‐height ratio.

^a^ These analyses were not performed in Zilioli et al. (2014) but we have conducted these analyses for complete clarity to the reader.

^b^ For the associations between total wins across weight categories for non‐Caucasian fighters, Zilioli et al. (2014) did not provide specific statistical details for each analysis but broadly note that, “These correlations were also not significant among non‐Caucasian fighters (average *r* = .27, lowest *p* = .090)” (p. 325).

#### 5.2.2 Exploratory analyses: Career stage and fWHR

As in Study 1, four moderation analyses were performed to examine the effect of debut date on the relationship between fWHRbrow and total fights, total wins, win percentage, win percentage controlling for total fights. Debut date did not significantly moderate the relationship between fWHRbrow and fight success (total fights: standardised interaction *B* = −0.01, *SE* = 0.03, *t* = −0.26, *p* = .80; total wins: standardised interaction *B* = −0.002, *SE* = 0.03, *t* = −0.04, *p* = .97; win percentage: standardised interaction *B* = 0.03, *SE* = 0.04, *t* = 0.76, *p* = .45; win percentage, controlling for total fights: standardised interaction *B* = 0.03, *SE* = 0.04, *t* = 0.72, *p* = .47).

#### 5.2.3 Extension of Zilioli et al. (2014)

Statistical associations between manual fWHR measurements and the 23 measures of fighting abilities are reported in Table 7. There was a significant positive association between fWHRbrow and lid and striking accuracy, such that fighters with larger fWHRs had greater striking accuracy, and a significant negative association between fWHRlid and wins by submission, where fighters with larger fWHRs were less likely to win by submission. While these effects disappeared after controlling for the relevant covariates (i.e., age, reach, leg reach, debut date, total fights, weight, and height), there were significant positive associations between fWHRbrow and landed takedowns and time‐adjusted landed takedowns, such that fighters with larger fWHRs were stronger grapplers, after controlling for these covariates. When we included the retired fighters, there were largely similar results (ESM). For interested readers, a correlation matrix including fWHR, win percentage, and all the covariates is included in the ESM. Of note, there was a significant, positive association between fWHR and weight.

**Table 7:** Correlations between manual fWHR and fighting abilities

	fWHR (eyelid)				fWHR (eyebrow)			
Outcome variable	Bivariate^a^		Partial^b^		Bivariate^a^		Partial^b^	
	*r*	*P*	*r*	*p*	*r*	*p*	*r*	*p*
Striking abilities								
Striking accuracy	**.13**	**.01**	.08	.12	**.12**	**.01**	.07	.19
Total strikes landed	−.04	.34	.05	.37	−.02	.64	.01	.86
Total strikes attempted	−.07	.14	.01	.76	−.04	.36	−.01	.80
Strikes landed per minute	.06	.24	.04	.47	.01	.81	.01	.93
Strikes landed in standing position	−.06	.24	.03	.57	−.05	.33	−.02	.75
Strikes landed in clinch position	−.02	.75	.05	.30	.02	.68	.04	.39
Strikes landed in ground position	−.01	.84	.05	.32	.03	.48	.06	.25
Strikes landed to the opponent's head	−.06	.22	.02	.68	−.03	.56	−.00	.94
Strikes landed to the opponent's body	−.03	.59	.07	.18	−.01	.85	.04	.48
Strikes landed to the opponent's legs	−.03	.57	.04	.38	−.03	.51	−.00	.94
Grappling abilities								
Grappling accuracy	.09	.07	.10	.07	.09	.07	.10	.06
Total takedowns landed	−.02	.70	.03	.59	.09	.07	**.14**	**.01**
Total takedowns attempted	−.05	.32	−.00	.99	.06	.21	.09	.07
Landed takedowns per 15 min	.04	.47	.03	.64	**.12**	**.01**	**.11**	**.04**
Attempted submissions per 15 min	.00	.94	−.03	.69	.04	.50	.02	.78
Defensive abilities								
Striking defense	−.05	.30	.01	.78	−.06	.17	−.00	.94
Takedown defense	−.05	.32	−.09	.10	−.04	.38	−.08	.12
Losses by KO/TKO	−.05	.28	−.03	.51	−.03	.52	−.06	.23
Losses by submission	−.03	.53	.02	.77	−.02	.62	−.00	.89
Losses by decision	−.07	.10	.00	.98	−.07	.11	−.05	.30
Fighting wins by type								
Wins by KO/TKO	.05	.23	.06	.27	.01	.82	−.05	.56
Wins by submission	−**.09**	**.04**	−.04	.42	.02	.60	.08	.13
Wins by decision	−.05	.30	.04	.46	.03	.51	.09	.06

*Note*: Statistically significant values have been bolded.

Abbreviations: fWHR, facial width‐to‐height ratio; KO/TKO, knockout/technical knockout.

^a^ Bivariate column represents the bivariate correlations between fWHR and each outcome variable.

^b^ Partial column represents the partial correlations between fWHR and each outcome variable, with age, reach, leg reach, debut date, total fights, weight, and height partialled out. Pairwise deletion was used. We considered it to be theoretically important to control for both age and debut date, as fighters can enter the UFC at a later age because they might come out of another professional organisation (e.g., NBA, NFL, WWE).

### 5.3 Discussion: Study 2

When we used manual measurements—and, therefore, methodology identical to Zilioli et al. (2014)—we successfully replicated their central results. We found significant results with almost identical effect sizes to the original study, for the relationship between fWHR and win percentage (1: win percentage; 2: win percentage, controlling for total fights; 3: win percentage, controlling for BMI; 4: win percentage, controlling for BMI and total fights). Indeed, for the relation between fWHR (manual, eyebrow) and win percentage, controlling for BMI and total fights, we found an effectively identical effect size to the original study (i.e., original study: *r* = .13; current study: *r* = .13). Our results suggest some error between automatic and manual measures, and research should ideally report both methods of measurement in fWHR research.

Consistent with the previous study, we failed to find support for the association between fWHR and blunt‐force trauma resistance. While we failed to find support for an association between fWHR and knockout power, there was a significant relationship between fWHR and body size. There was some support for a relationship between fWHR and aggression, with our strongest support being between fWHR and grappling ability (i.e., landed takedowns and time‐adjusted landed takedowns) which remained robust after controlling for demographic and allometric variables.

However, an additional limitation within Zilioli et al. (2014) was the use of fighter rather than fight data, which could be argued to violate independence of observations. It should be noted that fight data has been criticised (e.g., Richardson, 2020), as the winner of a single fight can be suddenly and unexpectedly determined which might make dichotomous fight outcome measures (i.e., win/lose) less than preferable. Indeed, much of human contest competition research has used fighter data (Aung et al., 2021; Richardson & Gilman, 2019; Richardson, 2020; Třebický et al., 2013, 2015, 2019). In Study 3, we sought to expand Zilioli et al. (2014) by examining the associations between fWHR and contest data among individual fighters. In so doing, our third study served as: (1) a conceptual replication of Zilioli et al. (2014) for the links between fWHR and fighting success; and (2) an extension, in that we further sought to examine the links between fWHR and aggression, blunt‐force resistance, and force output.

## 6 STUDY 3: FWHR AS A PREDICTOR OF INDIVIDUAL FIGHT DATA

### 6.1 Method: Study 3

Data were drawn from a publicly available dataset by Dabbert (2021). Similar to previous research (Lane & Briffa, 2020), this data had been scraped from ufcstats.com. There were 4566 unique fights and 1674 unique fighters who had participated in at least one UFC fight (*M*_*overall*_ = 5.46, *SD*_overall_ = 4.74; *M*_*focal*_ = 3.22, *SD*_focal_ = 2.64; *M*_*nonfocal*_ = 3.19, *SD*_*nonfocal*_ = 2.51) from March 21st, 2010, to February 6th, 2021. Fighters were assigned to be either blue or red fighters for a fight, but the fighter's color has been suggested to be associated with their abilities (Lane & Briffa, 2020). In line with Lane and Briffa (2020), we randomly assigned fighters to be either the focal or nonfocal fighter.

The current study used the focal and nonfocal fighters' height, reach, weight, and age; as well as fight‐specific information for the focal fighter's significant strikes landed, significant strikes attempted, striking accuracy, takedowns landed, takedowns attempted, and grappling accuracy. Focal outcome (coded as 0 = *focal fighter lost*; 1 = f*ocal fighter won*) and method of resolution (coded as 1 = *decision*; 2 = *knockout/technical knockout*; 3 = *submission*) data, the latter of which was used to examine the relationship between fWHR and blunt‐force resistance and force output (Lane & Briffa, 2020), was also present in the current dataset.

Using the names in Studies 1 and 2's fighter sample, we merged the automatic (eyebrow, eyelid) and manual (eyebrow, eyelid) fWHR measures, fighters' retirement/termination, debut date, leg reach, and total fights (i.e., all professional fights) to the respective focal and non‐focal fighter names in the individual fight dataset (names were checked for typographical errors before merging). Data merging was performed only for fighters who met the inclusion criteria for Studies 1 and 2, but now including retired fighters (retirement status was now added as a covariate for Study 3's analyses, as their inclusion did not substantially alter Studies 1 and 2's results to permit complete exclusion for Study 3). As in Studies 1 and 2, only male fighters who did not have nonneutral facial expressions, head tilts, or hair/beards that covered the zygions were merged. This resulted in 1367 unique fights where both focal and nonfocal fighter possessed both the automatic and manual fWHR measures—making this the largest individual fight dataset in an empirical paper to date (see Dixson et al., 2018; Lane & Briffa, 2020).

#### 6.1.1 Fighting success

Four generalized linear mixed‐effects models (LMMs)—one for each fWHR measurement (manual: eyebrow, eyelid; automatic: eyebrow, eyelid)—were conducted with a binomial error family to analyze the effect of focal fWHR on the focal outcome (i.e., focal win/loss). We controlled for the focal and nonfocal fighters' height, reach, weight, age, retirement status, debut date, leg reach, and total fights, as well as the nonfocal fighters' respective fWHR measurement. It should nonetheless be noted that inclusion of these covariates for our fighting success analyses did not affect results; our results are the same regardless of the inclusion of covariates. Similar to Lane and Briffa's (2020) method of analysis, we also included the method of resolution and the interaction between method of resolution and fWHR on the focal outcome. Model specification via backwards elimination was employed to gradually remove nonsignificant terms that improved the model fit (Akaike information criterion in lowest‐is‐best format) with analyses reported for the minimal adequate model. This is the same statistical process used by most animal contest research (Batchelor & Briffa, 2010, 2011; Batchelor et al., 2012; Hardy & Briffa, 2013; Lane & Briffa, 2020).

#### 6.1.2 Aggression

Twenty‐four LMMs—six for each fWHR measurement (manual: eyebrow, eyelid; automatic: eyebrow, eyelid)—with model specification via backwards elimination were conducted to examine the association between fWHR and an aggression‐related (i.e., Sell et al., 2012) outcome variable: (1) focal fighter's significant strikes landed; (2) significant strikes attempted; (3) striking accuracy; (4) takedowns landed; (5) takedowns attempted; and (6) grappling accuracy, controlling for those covariates mentioned in the previous section. Likewise, model specification via backwards elimination was employed and results were reported for the minimal adequate model. In line with Lane and Briffa (2020), the nonfocal fighter's corresponding aggression measurements were not controlled for because these metrics would likely be dependent on the focal fighter's behavior. In line with Lane and Briffa's (2020) methodology, we only used the focal fighter's aggression measurements and treated “fight” as the level of replication with random intercepts included to account for both focal and nonfocal fighters' IDs.

All analyses were carried out in RStudio using the package lme4 (Bates et al., 2015). For both fighting success and aggression analyses, variables were *Z*‐standardized before analysis and random intercepts were included to account for the ID of both focal and non‐focal fighters. For brevity, we reported the manual fWHR eyebrow measurement analyses here and all other fWHR measurement analyses (which exhibited the same pattern of results) in the ESM. In addition to our intercepts‐only models, we also included random slopes for both focal and nonfocal fighters' fWHR measurements; then, in another assortment of analyses, only focal fighters' fWHR measurements. Compared to the intercepts‐only analyses, equivalent intercepts‐and‐slopes models frequently resulted in a singular fit or a convergence error; nonetheless, intercepts‐and‐slopes models exhibited the same pattern of results as the intercepts‐only models. Yet, because singular fits are problematic for multilevel modelling, we reported the intercepts‐and‐slopes models in the ESM while the intercepts‐only models are reported in‐text. The R code and dataset for Study 3 are available on the OSF (https://osf.io/scde7/).

### 6.2 Results: Study 3

#### 6.2.1 Fighting success

Results showed no significant association between fWHRbrow (manual) and focal outcome (*β* = −0.09 ± 0.15, *χ* ^2^ = −0.56, *p* = .57), such that those with larger fWHRs were not significantly more likely to win the fight. There was also no significant interaction between fWHRbrow (manual) and the method of resolution on the focal outcome (*β* = 0.10 ± 0.09, *χ* ^2^ = 1.22, *p* = .22), such that those with larger fWHRs were not significantly more likely to win or lose via a specific strategy (i.e., via decision, submission, KO/TKO).

#### 6.2.2 Aggression

There were no significant associations between fWHRbrow (manual) and significant strikes landed (*β* = −0.01 ± 0.04, *t* = −0.39, 95% confidence interval [CI]: [−0.10, 0.07], *p* = .69), significant strikes attempted (*β* = −0.03 ± 0.04, *t* = −0.81, 95% CI: [−0.11, 0.05], *p* = 0.42), striking accuracy (*β* = 0.04 ± 0.04, *t* = 1.18, 95% CI: [−0.03, 0.11], *p* = .24), takedowns landed (*β* = 0.03 ± 0.04, *t* = 0.72, 95% CI: [−0.05, 0.11], *p* = .47), or takedowns attempted (*β* = 0.02 ± 0.05, *t* = 0.39, 95% CI: [−0.07, 0.11], *p* = .69). However, there was a significant association between fWHRbrow and grappling accuracy (*β* = 0.13 ± 0.04, *t* = 0.3.18, 95% CI: [0.05, 0.21], *p* = .002), such that those with larger fWHRs were more skilled grapplers. This latter effect was performed on the minimal adequate model (6 control variables) but was also significant in the most complex model with all 17 control variables (*β* = 0.14 ± 0.04, *t* = 3.29, 95% CI: [0.05, 0.22], *p* = .001) and also when there were no covariates (*β* = 0.07 ± 0.03, *t* = 2.46, 95% CI: [0.01, 0.13], *p* = .01). Strikingly, this relation between fWHR and grappling accuracy held across all three other fWHR measurements (ESM). These associations further held when random slopes were included (ESM).

## 7 GENERAL DISCUSSION

Zilioli et al. (2014) were among the first to show an association between male fWHR and physical aggression and fighting ability in professional mixed‐martial‐arts fighters, providing support for one of the leading explanations for why men with larger fWHRs show more aggression and antisocial behavior (Geniole et al., 2015; Haselhuhn et al., 2015). In the present work, we successfully replicated their main finding that the manual fWHRbrow measurement predicted men's fighting success (Study 2). We then successfully extended their work, finding associations between fWHR and grappling abilities, as a metric of aggression (Studies 1–3).

This association between fWHR and overall fighting success only held when we used Zilioli et al.'s (2014) original methodology (overall fighter data) and did not conceptually replicate when using fight‐specific data. This is consistent with previous critiques of using individual fight data, arguing that singular fights do not capture fighters' overall ability to succeed because singular fights can be suddenly and unexpectedly determined (Richardson, 2020). This supports the majority of research on human contest competition, which has elected to use overall fighter data (e.g., Aung et al., 2021; Richardson & Gilman, 2019; Richardson, 2020; Třebický et al., 2013, 2015, 2019). While individual fights would also be included in a fighter's win percentage, win percentage might: (1) better discriminate among fighters; and (2) more comprehensively capture fighters' *overall* RHP. For the latter, it should also be emphasised that ufc.com's overall fighter data comprises data spanning the entire UFC fighters' professional MMA career rather than solely UFC fight data, and thus would more comprehensively capture the fighters' overall RHP.

In our extension of the original study, there was generally minimal direct support for associations between fWHR and blunt‐force resistance (Carrier & Morgan, 2014) or force output (Sell et al., 2012; Zilioli et al., 2014). However, there was continual support for an association between fWHR and body size, which is consistent with the suggestion that the face is a cue to bodily features (Sell et al., 2009) which are, in turn, associated with force output and fighting success (Caton & Lewis, 2021a, 2021b). More directly, there was stronger support for the associations between fWHR and grappling abilities as a metric of aggression.

Studies 1–3 found continual support for the notion that men with larger fWHRs across all fWHR measurements possessed greater grappling abilities, even after controlling for demographic and allometric measurements. To explain why fWHR is specifically linked to grappling abilities, we contend that grappling, or the act of taking an opponent down to the ground, involves a more close‐combat, aggressive approach than does striking. Striking most often occurs in standing position and therefore at a distance (see descriptive statistics in the ESM). Because grappling uses more close combat strategies, grappling could be argued as a more aggressive approach because: (a) there is a higher likelihood of subsequent punches being landed (e.g., “ground and pound”); (b) there is a higher likelihood of now using elbows, fists, and knees to inflict damage; (c) landed strikes may be more damaging when in close quarters; (d) there is a reduced likelihood of escape for the one being struck; (e) there is a higher likelihood of using other methods to defeat their opponent other than strikes (e.g., submission holds). There are several potential mediating mechanisms for this link between fWHR and grappling‐based aggression; we discuss the role of testosterone, allometric scaling, and opponent intimidation.

### 7.1 Future research

#### 7.1.1 Potential mediators of fWHR and aggression

First, fWHR might be associated with aggressive outcomes due to its association with testosterone. However, links between fWHR and testosterone remain controversial (Bird et al., 2016; Whitehouse et al., 2015). This does not generalize to mean that masculine craniofacial morphology is not associated with testosterone levels; research has repeatedly shown that specific masculine facial features (e.g., large nose, jaw, chin) are associated with testosterone levels (Marečková et al., 2011; Roosenboom et al., 2018). Because fWHR is noted to share variance with these other androgen‐dependent facial cues (Dixson, 2018; Hodges‐Simeon et al., 2021; Zilioli et al., 2014), this shared variance could give rise to an association between fWHR and aggressive outcomes in fighters (Dixson, 2018). In line with the recommendations of recent research (e.g., Caton et al., in press; Dixson, 2018; Hodges‐Simeon et al., 2021), future research can rule out this alternative explanation by using multivariate geometric morphometric analyses.

Multivariate geometric morphometric (GMM) analyses are a statistical technique widely used in the biological sciences, validated in the 1980s and 1990s long before research began on fWHR (Adams & Otárola‐Castillo, 2013; Klingenberg, 2016). One advantage of these analyses is that they ensure multivariate normality (Klingenberg, 2016; Třebický et al., 2013). This allows researchers to make conclusions about the associations between bizygomatic width, independent of other facial metrics (e.g., jaw, chin, nose; Třebický et al., 2013). Another advantage of GMM analyses is that they algebraically remove allometry from stimuli (Adams & Otárola‐Castillo, 2013; Klingenberg, 2016). This is especially important considering that allometric scaling might have influenced the associations between fWHR and behavioral outcomes.

Another reason for why fWHR is associated with aggressive behavior is because fWHR may share variance with other bodily features more directly associated with fighting ability and aggression. There are three main methods to adjust for allometry: controlling for weight, height, or scaling stimuli to the same centroid size (Kleisner et al., 2021). The present work controlled for weight, height, arm span, and leg length. This could be argued to account for most of the variance associated with general size, and therefore account for variance associated with other anatomical features (e.g., arm span comprises both arm length and shoulder breadth, which are associated with fighting ability; Caton & Lewis, 2021a). Yet, there are much more statistically advanced methods to account for allometry more appropriately (Klingenberg, 2016). One such method is to scale facial stimuli to their centroid size, and thereby algebraically remove the influence of allometry (Klingenberg, 2016). Future research should examine the associations between facial shape and fighting ability using GMM analyses to better rule out the influence of allometry.

Another explanation for why fWHR is linked to within‐fight aggression is because fWHR acts as a threat display that intimidates rivals, increasing the chance of *successfully* executing aggressive manoeuvres against such rivals (e.g., grappling *accuracy*). Indeed, morphological features can evolve through sexual selection by acting as a threat display (e.g., beardedness; Dixson et al., 2018, 2021) and higher fWHRs broadcast threat (Geniole et al., 2015; Třebický et al., 2015; Zilioli et al., 2014). An opponent who feels threatened might underperform in combat, increasing the likelihood that higher fWHR men successfully execute aggressive manoeuvres against them.

#### 7.1.2 Considerations for reproducibility

Consistent with other research (Kosinski, 2017), the present work also showed some discrepancies between automatic and manual measurements. Automatic measurements are definitely invaluable for their speed in large samples (Jones et al., 2021) but some caution should be exercised when using automatic calculators that do not allow for the manual adjustment of misaligned landmarks (de Kok, 2017). Future research could employ automatic measurements that can be manually realigned to balance speed and accuracy (e.g., Webmorph). It would still be preferable to report both automatic (not manually realigned) and manual measurements for the purposes of scientific reproducibility, comprehensiveness, and to ensure the robusticity of results. If researchers can show that the same effect holds across all automatic and manual fWHR measurements involving both eyebrow and eyelid measurements (e.g., grappling accuracy; Study 3), then this would provide stronger support for their hypothesis.

With that said, research is encouraged to use the exact methodological and statistical methods used in the original study when conducting replications. Hidden moderator effects can lead to reproducibility concerns (Caton & Horan, 2021; Kenny & Judd, 2019) and we only successfully replicated Zilioli et al. (2014) when following their exact methodology: (1) examining the association between manual fWHR eyebrow measurements on (2) the most commonly used fighting success metric (win percentage) when using (3) the same sampling strategy (UFC fighters) in (4) overall fighter rather than fight‐specific data. When conducting replications then, researchers should prioritise direct over conceptual replications because any minor deviation in sampling, methodology, or statistical considerations can drive differences between an original study and its replication. When conceptual replications are used, researchers should *progressively* include deviations from the original study; if deviations are not progressively included, and the conceptual replication differs too much from the original study, then researchers will not know which specific deviation drove the differences in results.

Future research might wish to explore one minor deviation of the present work: examining the same effect in lower‐skilled fighting ecologies, where morphological structures should theoretically exhibit even stronger effects. Fighting skill can be conceptualized as the output of an evolved psychological system designed to motivate behaviors to overcome anatomically large (e.g., larger fWHR) opponents (Briffa & Lane, 2017). If larger anatomical structures evolutionarily increased fighting success, then fighting skill might have evolved as the output of a psychological system designed to motivate behaviors to overcome anatomically larger opponents (Briffa & Lane, 2017). Data from the UFC, a highly‐skilled fighting ecology, might show weaker effects between morphological structures and fighting success. Future research might find even stronger effects in less skilled fighting ecologies, particularly those without weight restrictions (e.g., Road Fighting Championship).

## 8 CONCLUSION

While much research implicates fWHR in a suite of behavioral outcomes (Geniole et al., 2015), associations between facial morphology and behavioral outcomes have been disputed (e.g., Kosinski, 2017; Todorov et al., 2015; Wang et al., 2019). One prominent explanation for why men with larger fWHRs show more antisocial behavior is predicated on the premise that facial structure is associated with fighting success (Craig et al., 2019; Dixson et al., 2021; Sell et al., 2009; Sell et al., 2016; Zilioli et al., 2014). The present work successfully replicated Zilioli et al.'s (2014) association between fWHR and fighting success, and successfully extended this work to show that men with larger fWHRs enact more aggressive strategies in real‐world fights. While future research will need to use geometric morphometric analyses to rule out alternative explanations and ensure the robusticity of results, the present work offers additional support for the argument that fWHR may have been shaped by sexual selection.

## Supporting information

Supporting information.

## ENDNOTES

^2^
Given that: (1) there have only been a limited number of draws in the UFC (i.e., approximately 40 out of over 5000 UFC fights; out of our sample of 520 fighters, only one fighter had been in three draws, eight fighters had been in two draws, and 54 fighters had been in one draw); (2) that the vast majority of UFC research has not accounted for draws in their research (e.g., Aung et al., 2021; Třebický et al., 2013, 2019; Zilioli et al., 2014); and that (3) draws might not be appropriate for capturing RHP (as an indicator of an organism's ability to win a fight), we decided not to account for draws in this data but rather to adopt the most commonly used measure of fight success (i.e., total wins divided by total fights).
