# Supplementary material for: Facial width‐to‐height ratio predicts fighting success: A direct replication and extension of Zilioli et al. (2014)
Source: Aggress Behav. 2022 Mar 8;48(5):449–65. doi: 10.1002/ab.22027 (PMC9544882; doi:10.1002/ab.22027)
Supplement: Supplementary file 1 — Supporting information. [file AB-48-449-s001.docx]

**Supplemental Material**

**Study 1: Automatic Measurements**

**Method**

**Aggression, Blunt Force Trauma Resistance, and Force Output**

***Aggression: Striking and Grappling Abilities***

Data were first collected on striking abilities; striking accuracy (*M* = 44.95; *SD* = 9.71; defined as significant strikes landed divided by significant strikes attempted), significant strikes landed (*M* = 291.82; *SD* = 284.62; defined as all distance strikes that land with some measure of force, plus power strikes in the clinch and on the ground), significant strikes attempted (*M* = 667.64; *SD* = 667.72; defined as all distance strikes attempted, plus power strikes attempted in the clinch and on the ground), significant strikes landed per minute (*M* = 3.57; *SD* = 1.57), significant strikes landed in a standing position (*M* = 204.43; *SD* = 212.93; defined as power strikes landed while opponents are standing at a distance), significant strikes landed in a clinch position (*M* = 40.52; *SD* = 46.37; defined as power strikes landed while opponents are standing at close range), significant strikes landed in a ground position (*M* = 48.33; *SD* = 59.67; defined as power strikes landed while opponents are on the ground), significant strikes landed to the opponent’s head (*M* = 190.02; *SD* = 188.52), significant strikes landed to the opponent’s body (*M* = 57.64; *SD* = 63.17), and significant strikes landed to the opponent’s legs (*M* = 44.73; *SD* = 53.87).

Data were then collected on grappling abilities; grappling accuracy (*M* = 41.67; *SD* = 22.87; defined as takedowns landed divided by takedowns attempted), takedowns landed (*M* = 9.69; *SD* = 11.70; defined as successful grappling manoeuvres that lead to control on the ground), takedowns attempted (*M* = 25.06; *SD* = 32.52; defined as grappling manoeuvres intended to lead to control on the ground), landed takedowns per 15 minutes (*M* = 1.75; *SD* = 1.56), and attempted submissions per 15 minutes (*M* = .92; *SD* = 1.03; defined as attempts to end the bout through grappling holds, chokes and joint locks).

***Blunt Force Trauma Resistance***

Data for fighters’ cumulative losses by knockout and technical knockout (*M* = 1.34; *SD* = 1.60)—as a proxy for blunt force trauma resistance—were collected from espn.com because ufc.com does not make this data available. Knockout losses are defined as a loss resulting from any strike or combination of strikes from the fighter’s opponent that leaves the fighter unable to continue, whereas technical knockout losses are defined as the referee’s termination of the fight due to the fighter being unable to intelligently defend themselves from strikes (Dixson et al., 2018). Fighters’ losses by knockout and technical knockout was collected as a single variable because espn.com does not separately provide a single cumulative number for fighters’ losses by knockout or technical knockout, but previous research has treated knockouts and technical knockouts as conceptually similar (Dixson et al., 2018).

***Force Output***

Data for fighters’ cumulative wins by knockout and technical knockout (*M* = 6.11; *SD* = 4.23)—as a proxy for knockout power and force output—were collected from ufc.com (which does not separate wins by knockout and wins by technical knockout but, again, previous research has treated knockouts and technical knockouts as conceptually similar; Dixson et al., 2018).

***Exploratory Variables***

Given that we have collected all available data from ufc.com and espn.com, we also included multiple additional variables that are related to fight performance and, potentially then, fWHR. These included striking defense (*M* = 56.11; *SD* = 9.72; defined as the percentage of the opponent’s strikes that did not land), takedown defense (*M* = 66.71; *SD* = 19.35; defined as the percentage of the opponent’s attempted takedowns that did not land), wins (*M* = 4.24; *SD* = 3.68) and losses by submission (*M* = 1.01; *SD* = 1.48; defined as a yield to the opponent, either because the fighter tapped out or because the fight was stopped by the referee or doctor [e.g., due to the fighter’s arm breaking in an armbar; the fighter passing out from a chokehold]), and wins (*M* = 4.03; *SD* = 3.25) and losses by decision (*M* = 2.12; *SD* = 2.14; defined as when the result of the bout does not end in knockout or submission, but is determined by the majority consensus of three judges).

**Results**

**Direct Replication of Zilioli et al. (2014)**

***fWHR Eyelid***

**Excluding retired fighters.** There were no statistically significant relationships between fWHRlid and total fights (*r* = -.02, *p* = .741), total wins (*r* = -.01, *p* = .830), or win percentage (*r* = .07, *p* = .117). There was also no significant relationship between win percentage and fWHRlid, controlling for the total number of fights (*r* = .07, *p* = .118). In line with Zilioli et al. (2014), we then controlled for BMI and found no significant relationships between fWHRlid and total fights (*r* = .00, *p* = .990), total wins (*r* = .01, *p* = .898), or win percentage (*r* = .06, *p* = .151). Finally, the relationship between fWHRlid and win percentage, while controlling for both BMI and the total number of fights, was not statistically significant (*r* = .07, *p* = .117).

Following Zilioli et al. (2014), we ran correlations between fWHRlid and fighting success within weight categories, within ethnicities and then among fighters of different ethnicities within each weight category. The weight categories and sample sizes were as follows; in lightweight (from 57 to 70kg, *n* = 265), middleweight (from 77 to 84kg, *n* = 147), and heavyweight (from 90kg to 120kg, *n* = 108) fighters. When restricting analyses to weight categories, there were no statistically significant relationships between fWHRlid and total fights (lightweight: *r* = -.05, *p* = .39; middleweight: *r* = .04, p = .62; heavyweight fighters: *r* = .01, *p* = .88), total wins (lightweight: *r* = -.05, *p* = .406; middleweight: *r* = .06, *p* = .468; heavyweight fighters: *r* = .01, *p* = .916), win percentage (lightweight: *r* = .02, *p* = .69; middleweight: *r* = .07, *p* = .41; heavyweight fighters: *r* = .13, *p* = .18), or win percentage controlling for total fights (lightweight: *r* = .00, *p* = .96; middleweight: *r* = .10, *p* = .23; heavyweight fighters: *r* = .14, *p* = .15).

We then restricted analyses to ethnicity. In Caucasian fighters (*n* = 353), fWHRlid was not significantly correlated with total wins (*r* = -.03, *p* = .576), total fights (*r* = -.03, *p* = .552), win percentage (*r* = .06, *p* = .242), or win percentage controlling for total fights (*r* = .05, *p* = .311). In non-Caucasian fighters (*n* = 167), fWHRlid was also not significantly correlated with total wins (*r* = .03, *p* = .721), total fights (*r* = .02, *p* = .830), win percentage (*r* = .09, *p* = .246), or win percentage controlling for total fights (*r* = .10, *p* = .189). Within Caucasian fighters, there were no significant associations between fWHRlid and total fights (lightweight: *r* = -.08, *p* = .33; middleweight: *r* = -.05, p = .63; heavyweight fighters: *r* = .07, *p* = .58), total wins (lightweight: *r* = -.08, *p* = .30; middleweight: *r* = -.04, *p* = .72; heavyweight fighters: *r* = .06, *p* = .61), win percentage (lightweight: *r* = .05, *p* = .50; middleweight: *r* = .04, *p* = .69; heavyweight fighters: *r* = .12, *p* = .33), or win percentage controlling for total fights (lightweight: *r* = .02, *p* = .80; middleweight: *r* = .02, *p* = .86; heavyweight fighters: *r* = .14, *p* = .27). Likewise, within non-Caucasian fighters there were no significant relationships between fWHRlid and total fights (lightweight: *r* = -.03, *p* = .81; middleweight: *r* = .29, p = .08; heavyweight fighters: *r* = -.06, *p* = .73), total wins (lightweight: *r* = -.01, *p* = .90; middleweight: *r* = .31, *p* = .06; heavyweight fighters: *r* = -.06, *p* = .72), win percentage (lightweight: *r* = .00, *p* = .98; middleweight: *r* = .15, *p* = .36; heavyweight fighters: *r* = .14, *p* = .39), or win percentage controlling for total fights (lightweight: *r* = -.00, *p* = .96; middleweight: *r* = .27, *p* = .11; heavyweight fighters: *r* = .13, *p* = .44).

**Including retired fighters.** There were no statistically significant relationships between fWHRlid and total fights (*r* = -.01, *p* = .80), total wins (*r* = -.01, *p* = .90), or win percentage (*r* = .07, *p* = .09). There was also no significant relationship between win percentage and fWHRlid, controlling for the total number of fights (*r* = .07, *p* = .08). In line with Zilioli et al. (2014), we then controlled for BMI and found no significant relationships between fWHRlid and total fights (*r* = -.00, *p* = .98), total wins (*r* = .00, *p* = .93), or win percentage (*r* = .07, *p* = .12). Finally, the relationship between fWHRlid and win percentage, while controlling for both BMI and the total number of fights, was not statistically significant (*r* = .07, *p* = .90).

Following Zilioli et al. (2014), we ran correlations between fWHRlid and fighting success within weight categories, within ethnicities and then among fighters of different ethnicities within each weight category. When restricting analyses to weight categories, there were no statistically significant relationships between fWHRlid and total fights (lightweight: *r* = -.02, *p* = .76; middleweight: *r* = -.00, p = .98; heavyweight fighters: *r* = .01, *p* = .90), total wins (lightweight: *r* = -.02, *p* = .76; middleweight: *r* = .02, *p* = .78; heavyweight fighters: *r* = .01, *p* = .92), win percentage (lightweight: *r* = .02, *p* = .76; middleweight: *r* = .01, *p* = .20; heavyweight fighters: *r* = .12, *p* = .19), or win percentage controlling for total fights (lightweight: *r* = .01, *p* = .85; middleweight: *r* = .11, *p* = .16; heavyweight fighters: *r* = .13, *p* = .16).

We then restricted analyses to ethnicity. In Caucasian fighters, fWHRlid was not significantly correlated with total wins (*r* = -.03, *p* = .54), total fights (*r* = -.04, *p* = .64), win percentage (*r* = .09, *p* = .265), or win percentage controlling for total fights (*r* = .06, *p* = .22). In non-Caucasian fighters, fWHRlid was also not significantly correlated with total wins (*r* = .03, *p* = .70), total fights (*r* = .04, *p* = .64), win percentage (*r* = .09, *p* = .27), or win percentage controlling for total fights (*r* = .10, *p* = .18). Within Caucasian fighters, there were no significant associations between fWHRlid and total fights (lightweight: *r* = -.07, *p* = .37; middleweight: *r* = -.07, p = .42; heavyweight fighters: *r* = .09, *p* = .42), total wins (lightweight: *r* = -.07, *p* = .32; middleweight: *r* = -.05, *p* = .54; heavyweight fighters: *r* = .09, *p* = .44), win percentage (lightweight: *r* = .05, *p* = .50; middleweight: *r* = .08, *p* = .37; heavyweight fighters: *r* = .10, *p* = .38), or win percentage controlling for total fights (lightweight: *r* = .02, *p* = .79; middleweight: *r* = .05, *p* = .58; heavyweight fighters: *r* = .12, *p* = .29). Likewise, within non-Caucasian fighters there were no significant relationships between fWHRlid and total fights (lightweight: *r* = .04, *p* = .70; middleweight: *r* = .24, p = .13; heavyweight fighters: *r* = -.11, *p* = .51), total wins (lightweight: *r* = .04, *p* = .70; middleweight: *r* = .26, *p* = .10; heavyweight fighters: *r* = -.11, *p* = .50), win percentage (lightweight: *r* = -.01, *p* = .90; middleweight: *r* = .15, *p* = .33; heavyweight fighters: *r* = .16, *p* = .34), or win percentage controlling for total fights (lightweight: *r* = -.00, *p* < .99; middleweight: *r* = .23, *p* = .15; heavyweight fighters: *r* = .12, *p* = .46).

***fWHR Eyebrow***

**Including retired fighters.** There were no statistically significant relationships between fWHRbrow and total fights (*r* = .03, *p* = .48), total wins (*r* = .04, *p* = .35), or win percentage (*r* = .07, *p* = .11). There was also no significant relationship between win percentage and fWHRbrow, controlling for the total number of fights (*r* = .07, *p* = .08). In line with Zilioli et al. (2014), we then controlled for BMI and found no significant relationships between fWHRbrow and total fights (*r* = -.00, *p* = .98), total wins (*r* = .00, *p* = .93), or win percentage (*r* = .07, *p* = .12). Finally, the relationship between fWHRbrow and win percentage, while controlling for both BMI and the total number of fights, was not statistically significant (*r* = .07, *p* = .09).

Following Zilioli et al. (2014), we ran correlations between fWHRbrow and fighting success within weight categories, within ethnicities and then among fighters of different ethnicities within each weight category. When restricting analyses to weight categories, there were no statistically significant relationships between fWHRbrow and total fights (lightweight: *r* = .05, *p* = .42; middleweight: *r* = .02, p = .80; heavyweight fighters: *r* = .04, *p* = .68), total wins (lightweight: *r* = .06, *p* = .34; middleweight: *r* = .03, *p* = .74; heavyweight fighters: *r* = .05, *p* = .56), win percentage (lightweight: *r* = .05, *p* = .40; middleweight: *r* = .05, *p* = .53; heavyweight fighters: *r* = .10, *p* = .28), or win percentage controlling for total fights (lightweight: *r* = .01, *p* = .85; middleweight: *r* = .11, *p* = .16; heavyweight fighters: *r* = .13, *p* = .16).

We then restricted analyses to ethnicity. In Caucasian fighters (*n* = 353), fWHRbrow was not significantly correlated with total wins (*r* = .01, *p* = .86), total fights (*r* = .01, *p* = .84), win percentage (*r* = .04, *p* = .47), or win percentage controlling for total fights (*r* = .06, *p* = .22). In non-Caucasian fighters (*n* = 167), fWHRbrow was also not significantly correlated with total wins (*r* = .07, *p* = .37), total fights (*r* = .09, *p* = .25), win percentage (*r* = .10, *p* = .19), or win percentage controlling for total fights (*r* = .10, *p* = .18). Within Caucasian fighters, there were no significant associations between fWHRbrow and total fights (lightweight: *r* = -.02, *p* = .79; middleweight: *r* = -.07, p = .46; heavyweight fighters: *r* = .13, *p* = .24), total wins (lightweight: *r* = -.01, *p* = .94; middleweight: *r* = -.06, *p* = .48; heavyweight fighters: *r* = .15, *p* = .20), win percentage (lightweight: *r* = .02, *p* = .75; middleweight: *r* = .04, *p* = .67; heavyweight fighters: *r* = .07, *p* = .57), or win percentage controlling for total fights (lightweight: *r* = .02, *p* = .79; middleweight: *r* = .05, *p* = .58; heavyweight fighters: *r* = .12, *p* = .29). Likewise, within non-Caucasian fighters there were no significant relationships between fWHRbrow and total fights (lightweight: *r* = .12, *p* = .26; middleweight: *r* = .26, p = .09; heavyweight fighters: *r* = -.09, *p* = .60), total wins (lightweight: *r* = .05, *p* = .15; middleweight: *r* = .26, *p* = .09; heavyweight fighters: *r* = -.08, *p* = .64), win percentage (lightweight: *r* = .05, *p* = .63; middleweight: *r* = .06, *p* = .68; heavyweight fighters: *r* = .17, *p* = .29), or win percentage controlling for total fights (lightweight: *r* = -.00, *p* > .99; middleweight: *r* = .23, *p* = .15; heavyweight fighters: *r* = .12, *p* = .46).

**Exploratory Analyses: Career Stage and fWHRlid**

Using Hayes’ (2013) SPSS PROCESS macro (model 1; v.3.5; 10000 bootstrap samples; 95% bias-corrected confidence intervals) then, four moderation analyses were performed to examine the effect of the moderating variable (i.e., debut date) on the relationship between a predictor (i.e., fWHRlid) and an outcome variable (i.e., total fights, total wins, win percentage, win percentage controlling for total fights). Debut date did not significantly moderate the relationship between fWHRlid and fight success (total fights: standardised interaction *B* = -.04, *SE* = .03, *t* = -1.32, *p* = .19; total wins: standardised interaction *B* = -.05, *SE* = .03, *t* = -1.42, *p* = .16; win percentage: standardised interaction *B* = .01, *SE* = .04, *t* = .20, *p* = .84; win percentage, controlling for total fights: standardised interaction *B* = -.01, *SE* = .04, *t* = -.12, *p* = .90).

**Extension of Zilioli et al. (2014)**

***Including Retired Fighters***

Table S1 presents the correlations between fWHR and aggression, blunt-force trauma resistance, and force output. There was a significant positive correlation between fWHRbrow and time-adjusted landed takedowns.

**Table S1.** Correlations between fWHR and fighting abilities. Retired fighters included.

|  | fWHR (Eyelid) | | | | | |  | | fWHR (Eyebrow) | | | | | | | | | | |
| --- | --- | --- | --- | --- | --- | --- | --- | --- | --- | --- | --- | --- | --- | --- | --- | --- | --- | --- | --- |
| Outcome Variable | Bivariate^1^ | |  | Partial^2^ | | |  | | Bivariate^1^ | | | |  | | Partial^2^ | | |  |  |
|  | *r* | *p* |  | *r* | *p* | |  | | *r* | | *p* | |  | | *r* | | *p* | | |
| *Striking Abilities* |  |  |  |  |  |  | |  | |  | |  | |  | |  | | |  |
| Striking accuracy | .04 | .419 |  | -.00 | .954 |  | | .04 | | .325 | |  | | .00 | | .698 | | |  |
| Total strikes landed | .02 | .713 |  | .06 | .251 |  | | .02 | | .661 | |  | | .03 | | .498 | | |  |
| Total strikes attempted | .01 | .904 |  | .05 | .359 |  | | .01 | | .843 | |  | | .03 | | .614 | | |  |
| Strikes landed per minute | .01 | .853 |  | -.01 | .895 |  | | .01 | | .881 | |  | | -.00 | | .945 | | |  |
| Strikes landed in standing position | .03 | .564 |  | .07 | .174 |  | | .02 | | .648 | |  | | .04 | | .454 | | |  |
| Strikes landed in clinch position | -.01 | .790 |  | .00 | .962 |  | | .01 | | .820 | |  | | .01 | | .853 | | |  |
| Strikes landed in ground position | -.01 | .879 |  | .01 | .817 |  | | .00 | | .958 | |  | | .01 | | .922 | | |  |
| Strikes landed to the opponent’s head | .02 | .723 |  | .05 | .304 |  | | .02 | | .708 | |  | | .03 | | .608 | | |  |
| Strikes landed to the opponent’s body | .00 | .983 |  | .04 | .470 |  | | .01 | | .766 | |  | | .03 | | .563 | | |  |
| Strikes landed to the opponent’s legs | .02 | .654 |  | .05 | .269 |  | | .01 | | .759 | |  | | .03 | | .573 | | |  |
| *Grappling Abilities* |  |  |  |  |  |  | |  | |  | |  | |  | |  | | |  |
| Grappling accuracy | .04 | .437 |  | .05 | .374 |  | | .04 | | .455 | |  | | .04 | | .420 | | |  |
| Total takedowns landed | .04 | .425 |  | .07 | .170 |  | | .05 | | .309 | |  | | .08 | | 142 | | |  |
| Total takedowns attempted | .01 | .843 |  | .03 | .537 |  | | .02 | | .735 | |  | | .03 | | .575 | | |  |
| Landed takedowns per 15 minutes | .06 | .242 |  | .05 | .380 |  | | **.11** | | **.024** | |  | | **.10** | | **.041** | | |  |
| Attempted submissions per 15 minutes | .00 | .953 |  | -.03 | .641 |  | | .07 | | .177 | |  | | .05 | | .382 | | |  |
| *Defensive Abilities* |  |  |  |  |  |  | |  | |  | |  | |  | |  | | |  |
| Striking defence | -.03 | .496 |  | .01 | .811 |  | | -.04 | | .403 | |  | | .00 | | .935 | | |  |
| Takedown defence | -.04 | .440 |  | -.06 | .275 |  | | -.02 | | .753 | |  | | -.03 | | .583 | | |  |
| Losses by KO and TKO | -.01 | .777 |  | -.02 | .701 |  | | .01 | | .893 | |  | | -.03 | | .520 | | |  |
| Losses by submission | -.06 | .185 |  | -.06 | .232 |  | | -.01 | | .612 | |  | | -.04 | | .360 | | |  |
| Losses by decision | .00 | .954 |  | .03 | .557 |  | | .00 | | .932 | |  | | -.00 | | .947 | | |  |
| *Fighting Wins by Type* |  |  |  |  |  |  | |  | |  | |  | |  | |  | | |  |
| Wins by KO and TKO | .03 | .421 |  | -.00 | .954 |  | | .03 | | .512 | |  | | .07 | | .530 | | |  |
| Wins by submission | -.03 | .551 |  | -.03 | .591 |  | | .07 | | .090 | |  | | .07 | | .162 | | |  |
| Wins by decision | .00 | .947 |  | .02 | .740 |  | | .03 | | .429 | |  | | .04 | | .380 | | |  |

*Note.* ^1^Bivariate column represents the bivariate correlations between fWHR and each outcome variable. ^2^Partial column represents the partial correlations between fWHR and each outcome variable, with age, reach, leg reach, debut date, total fights, weight, and height partialled out. Pairwise deletion was used.

Table S2 presents the correlations between all fWHR measurements, fighting success, and the covariates that were controlled for in the previous table, excluding retired fighters.

**Table S2.** Correlations between fWHR measurements, fighting success, and covariates, excluding retired fighters.

| fWHR Measure | 1 | 2 | 3 | 4 | 5 | 6 | 7 | 8 |
| --- | --- | --- | --- | --- | --- | --- | --- | --- |
| fWHR (Automatic, Eyelid) | .07 | -.01 | .04 | -.03 | .04 | -.02 | .15*** | .05 |
| fWHR (Automatic, Eyebrow) | .06 | .03 | .04 | .01 | -.00 | .04 | .16*** | .07 |
| fWHR (Manual, Eyelid) | .10* | -.05 | .08 | -.02 | .11* | -.08 | .18*** | .05 |
| fWHR (Manual, Eyebrow) | .12** | .02 | .04 | .00 | .05 | -.03 | .22*** | .05 |
| Outcome | 1 | 2 | 3 | 4 | 5 | 6 | 7 | 8 |
| 1. Fight Success | - |  |  |  |  |  |  |  |
| 1. Age | -.32*** | - |  |  |  |  |  |  |
| 1. Reach | .03 | .05 | - |  |  |  |  |  |
| 1. Leg Reach | .01 | .10* | .68*** | - |  |  |  |  |
| 1. Debut | .35*** | -.62*** | .01 | -.04 | - |  |  |  |
| 1. Total Fights | -.40*** | .49*** | -.06 | -.02 | -.68*** | - |  |  |
| 1. Weight | .03 | .13** | .73*** | .60*** | .02 | -.10* | - |  |
| 1. Height | -.01 | .03 | .88*** | .71*** | .01 | -.066 | .76*** | - |

*Note.* * p < .05. ** p < .01. *** p < .001.

Table S3 presents the correlations between all fWHR measurements, fighting success, and the covariates that were controlled for in Table S1, including retired fighters.

**Table S3.** Correlations between fWHR measurements, fighting success, and covariates, including retired fighters.

| fWHR Measure | 1 | 2 | 3 | 4 | 5 | 6 | 7 | 8 |
| --- | --- | --- | --- | --- | --- | --- | --- | --- |
| fWHR (Automatic, Eyelid) | .07 | -.04 | .03 | -.03 | .06 | -.01 | .12** | .04 |
| fWHR (Automatic, Eyebrow) | .07 | -.01 | .03 | .01 | .03 | .03 | .14* | .06 |
| fWHR (Manual, Eyelid) | .12** | -.09* | .08 | -.02 | .13** | -.08 | .17*** | .05 |
| fWHR (Manual, Eyebrow) | .14** | -.02 | .04 | .00 | .08 | -.03 | .21*** | .05 |
| Outcome | 1 | 2 | 3 | 4 | 5 | 6 | 7 | 8 |
| 1. Fight Success | - |  |  |  |  |  |  |  |
| 1. Age | -.35*** | - |  |  |  |  |  |  |
| 1. Reach | .02 | .09* | - |  |  |  |  |  |
| 1. Leg Reach | -.00 | .11* | .69*** | - |  |  |  |  |
| 1. Debut | .37*** | -.68*** | -.03 | .05 | - |  |  |  |
| 1. Total Fights | -.40*** | .50*** | -.05 | -.02 | -.68*** | - |  |  |
| 1. Weight | .03 | .15*** | .74*** | .60*** | -.02 | -.08 | - |  |
| 1. Height | -.01 | .05 | .88*** | .71*** | -.01 | -.06 | .76*** | - |

*Note.* * p < .05. ** p < .01. *** p < .001.

**Study 2: Manual Measurements**

**Results**

**Direct Replication of Zilioli et al. (2014)**

***fWHR Eyebrow***

**Including retired fighters.** There were no statistically significant relationships between fWHRbrow and total fights (*r* = -.03, *p* = .47), total wins (*r* = .00, *p* = .99), but there was a significant association with win percentage (*r* = .14, *p* = .001). There was no significant relationship between win percentage and fWHRbrow, controlling for the total number of fights (*r* = .07, *p* = .08). In line with Zilioli et al. (2014), we then controlled for BMI and found no significant relationships between fWHRbrow and total fights (*r* = -.00, *p* = .98), total wins (*r* = .00, *p* = .93), or win percentage (*r* = .07 *p* = .12). Finally, the relationship between fWHRbrow and win percentage, while controlling for both BMI and the total number of fights, was not statistically significant (*r* = .07, *p* = .09).

Following Zilioli et al. (2014), we ran correlations between fWHRbrow and fighting success within weight categories, within ethnicities and then among fighters of different ethnicities within each weight category. When restricting analyses to weight categories, there were no statistically significant relationships between fWHRbrow and total fights (lightweight: *r* = -.04, *p* = .56; middleweight: *r* = .11, p = .16; heavyweight fighters: *r* = -.01, *p* = .89), total wins (lightweight: *r* = -.02, *p* = .78; heavyweight fighters: *r* = .02, *p* = .84), win percentage (lightweight: *r* = .11, *p* = .07; middleweight: *r* = .13, *p* = .09; heavyweight fighters: *r* = .14, *p* = .12), or win percentage controlling for total fights (lightweight: *r* = .01, *p* = .85;middleweight fighters (*r* = .11, *p* = .16), or heavyweight fighters: *r* = .13, *p* = .16). There was a significant association between fWHR and total wins in middleweight fighters (*r* = .15, *p* = .045).

We then restricted analyses to ethnicity. In Caucasian fighters, fWHRbrow was not significantly correlated with total wins (*r* = -.02, *p* = .75), total fights (*r* = .02, *p* = .68), ), but was significantly correlated with win percentage (*r* = .13, *p* = .01), but not win percentage controlling for total fights (*r* = .06, *p* = .22). In non-Caucasian fighters, fWHRbrow was also not significantly correlated with total wins (*r* = -.06, *p* = .42), total fights (*r* = -.05, *p* = .55), but was significantly correlated with win percentage (*r* = .16, *p* = .04) but not win percentage controlling for total fights (*r* = .10, *p* = .18). Within Caucasian fighters, there were no significant associations between fWHRbrow and total fights (lightweight: *r* = -.03, *p* = .67; middleweight: *r* = .10, p = .28; heavyweight fighters: *r* = .02, *p* = .84), total wins (lightweight: *r* = -.03, *p* = .70; middleweight: *r* = .16, *p* = .08; heavyweight fighters: *r* = .07, *p* = .52), win percentage (lightweight: *r* = .08, *p* = .31; middleweight: *r* = .15, *p* = .10; heavyweight fighters: *r* = .14, *p* = .21), or win percentage controlling for total fights (lightweight: *r* = .02, *p* = .76; middleweight fighters (*r* = .05, *p* = .56), or heavyweight fighters: *r* = .12, *p* = .23). Within non-Caucasian fighters, there were no significant relationships between fWHRbrow and total fights (lightweight: *r* = -.03, *p* = .76; middleweight: *r* = .16, p = .32; heavyweight fighters: *r* = -.07, *p* = .66), total wins (lightweight: *r* = .01, *p* = .95; middleweight: *r* = .14, *p* = .36; heavyweight fighters: *r* = -.07, *p* = .66), win percentage (lightweight: *r* = .15, *p* = .17; middleweight: *r* = .05, *p* = .74; heavyweight fighters: *r* = .15, *p* = .37), or win percentage controlling for total fights (lightweight: *r* = -.00, *p* = .99; middleweight: *r* = .23, *p* = .15; heavyweight fighters: *r* = .12, *p* = .46).

***fWHR Eyelid***

**Excluding retired fighters.** There were no statistically significant relationships between fWHRlid and total fights (*r* = -.08, *p* = .06) or total wins (*r* = -.07, *p* = .10), but there was a significant relationship between fWHRlid and win percentage (*r* = .10, *p* = .02). However, there was no significant relationship between win percentage and fWHRlid, when controlling for the total number of fights (*r* = .07, *p* = .10). In line with Zilioli et al. (2014), we then controlled for BMI and found no significant relationships between fWHRlid and total fights (*r* = -.07, *p* = .13), total wins (*r* = -.06, *p* = .20), but there was a significant relationship between fWHRlid and win percentage (*r* = .09, *p* = .03). Finally, the relationship between fWHRlid and win percentage, while controlling for both BMI and the total number of fights, was not statistically significant (*r* = .07, *p* = .09).

Following Zilioli et al. (2014), we ran correlations between fWHRlid and fighting success within weight categories, within ethnicities and then among fighters of different ethnicities within each weight category. When restricting analyses to weight categories, there were no statistically significant relationships between fWHRlid and total fights (lightweight: *r* = -.11, *p* = .08; middleweight: *r* = .04, p = .67; heavyweight fighters: *r* = -.03, *p* = .72), total wins (lightweight: *r* = -.10, *p* = .10; middleweight: *r* = .05, *p* = .55; heavyweight fighters: *r* = -.02, *p* = .84), win percentage (lightweight: *r* = .05, *p* = .40; middleweight: *r* = .08, *p* = .31; heavyweight fighters: *r* = .16, *p* = .10), or win percentage controlling for total fights (lightweight: *r* = .89, *p* = .26; middleweight: *r* = .11, *p* = .17; heavyweight fighters: *r* = .16, *p* = .10).

We then restricted analyses to ethnicity. In Caucasian fighters, fWHRlid was not significantly correlated with total wins (*r* = -.05, *p* = .38), total fights (*r* = -.06, *p* = .23), win percentage (*r* = .10, *p* = .06), or win percentage controlling for total fights (*r* = .08, *p* = .13). In non-Caucasian fighters, fWHRlid was also not significantly correlated with total wins (*r* = -.11, *p* = .15), total fights (*r* = -.12, *p* = .12), win percentage (*r* = .14, *p* = .07), or win percentage controlling for total fights (*r* = .11, *p* = .17). Within Caucasian fighters, there were no significant associations between fWHRlid and total fights (lightweight: *r* = -.09, *p* = .22; middleweight: *r* = .03, *p* = .77; heavyweight fighters: *r* = -.02, *p* = .90), total wins (lightweight: *r* = -.09, *p* = .22; middleweight: *r* = .04, *p* = .65; heavyweight fighters: *r* = .03, *p* = .80), win percentage (lightweight: *r* = .06, *p* = .46; middleweight: *r* = .05, *p* = .60; heavyweight fighters: *r* = .21, *p* = .09), or win percentage controlling for total fights (lightweight: *r* = .01, *p* = .85; middleweight: *r* = .08, *p* = .43; heavyweight fighters: *r* = .21, *p* = .09). Likewise, within non-Caucasian fighters there were no significant relationships between fWHRlid and total fights (lightweight: *r* = -.15, *p* = .16; middleweight: *r* = .04, p = .83; heavyweight fighters: *r* = -.03, *p* = .87), total wins (lightweight: *r* = -.12, *p* = .25; middleweight: *r* = .04, *p* = .80; heavyweight fighters: *r* = -.04, *p* = .80), win percentage (lightweight: *r* = .10, *p* = .34; middleweight: *r* = .19, *p* = .26; heavyweight fighters: *r* = .09, *p* = .61), or win percentage controlling for total fights (lightweight: *r* = .06, *p* = .57; middleweight: *r* = .21, *p* = .21; heavyweight fighters: *r* = .08, *p* = .63).

**Including retired fighters.** There were no statistically significant relationships between fWHRlid and total fights (*r* = -.03, *p* = .47), total wins (*r* = .00, *p* = .99), but was significant for win percentage (*r* = .14, *p* = .001). There was also no significant relationship between win percentage and fWHRlid, controlling for the total number of fights (*r* = .07, *p* = .08). In line with Zilioli et al. (2014), we then controlled for BMI and found no significant relationships between fWHRlid and total fights (*r* = -.00, *p* = .98), total wins (*r* = .00, *p* = .93), or win percentage (*r* = .07, *p* = .12). Finally, the relationship between fWHRlid and win percentage, while controlling for both BMI and the total number of fights, was not statistically significant (*r* = .07, *p* = .90).

Following Zilioli et al. (2014), we ran correlations between fWHRlid and fighting success within weight categories, within ethnicities and then among fighters of different ethnicities within each weight category. When restricting analyses to weight categories, there were no statistically significant relationships between fWHRlid and total fights (lightweight: *r* = -.04, *p* = .56; middleweight: *r* = .11, p = .16; heavyweight fighters: *r* = -.01, *p* = .90), total wins (lightweight: *r* = -.02, *p* = .78; heavyweight fighters: *r* = .02, *p* = .83), win percentage (lightweight: *r* = .11, *p* = .07; middleweight: *r* = .13, *p* = .09; heavyweight fighters: *r* = .14, *p* = .12), or win percentage controlling for total fights (lightweight: *r* = .01, *p* = .85; middleweight: *r* = .09, *p* = .22; heavyweight fighters: *r* = .13, *p* = .17). There was a significant association between fWHR and total wins in middleweight fighters (*r* = .15, *p* = .045).

We then restricted analyses to ethnicity. In Caucasian fighters, fWHRlid was not significantly correlated with total wins (*r* = -.02, *p* = .75), or total fights (*r* = .02, *p* = .68), but was significant for win percentage (*r* = .13, *p* = .01), but not win percentage controlling for total fights (*r* = .06, *p* = .22). In non-Caucasian fighters, fWHRlid was also not significantly correlated with total wins (*r* = -.06, *p* = .42), or total fights (*r* = -.05, *p* = .55), but was significant for win percentage (*r* = .16, *p* = .035), but not win percentage controlling for total fights (*r* = .10, *p* = .18). Within Caucasian fighters, there were no significant associations between fWHRlid and total fights (lightweight: *r* = -.03, *p* = .67; middleweight: *r* = .10, p = .28; heavyweight fighters: *r* = .02, *p* = .84), total wins (lightweight: *r* = -.03, *p* = .70; middleweight: *r* = .16, *p* = .08; heavyweight fighters: *r* = .07, *p* = .52), win percentage (lightweight: *r* = .08, *p* = .31; middleweight: *r* = .15, *p* = .10; heavyweight fighters: *r* = .14, *p* = .21), or win percentage controlling for total fights (lightweight: *r* = .02, *p* = .79; middleweight: *r* = .05, *p* = .58; heavyweight fighters: *r* = .12, *p* = .29). Likewise, within non-Caucasian fighters there were no significant relationships between fWHRlid and total fights (lightweight: *r* = -.03, *p* = .76; middleweight: *r* = .16, p = .32; heavyweight fighters: *r* = .15, *p* = .37), total wins (lightweight: *r* = .01, *p* = .95; middleweight: *r* = .14, *p* = .36; heavyweight fighters: *r* = -.07, *p* = .66), win percentage (lightweight: *r* = .15, *p* = .17; middleweight: *r* = .05, *p* = .74; heavyweight fighters: *r* = .15, *p* = .37), or win percentage controlling for total fights (lightweight: *r* = -.00, *p* = .99; middleweight: *r* = .23, *p* = .15; heavyweight fighters: *r* = .12, *p* = .46).

**Exploratory Analyses: Career Stage and fWHR**

***fWHR eyelid***

Four moderation analyses were performed to examine the effect of the moderating variable (i.e., debut date) on the relationship between a predictor (i.e., fWHRlid) and an outcome variable (i.e., total fights, total wins, win percentage, win percentage controlling for total fights). Debut date did not significantly moderate the relationship between fWHRlid and fight success (total fights: standardised interaction *B* = -.01, *SE* = .03, *t* = -.38, *p* = .70; total wins: standardised interaction *B* = -.02, *SE* = .03, *t* = -.58, *p* = .56; win percentage: standardised interaction *B* = .001, *SE* = .04, *t* = .02, *p* = .98; win percentage, controlling for total fights: standardised interaction *B* = -.003, *SE* = .04, *t* = -.07, *p* = .95).

**Extension of Zilioli et al. (2014)**

***Including Retired Fighters***

Table S4 presents the correlations between fWHR and aggression, blunt-force trauma resistance, and force output.

**Table S4.** Correlations between manual fWHR and fighting abilities, with retired fighters.

|  | fWHR (Eyelid) | | | | |  | fWHR (Eyebrow) | | | | |
| --- | --- | --- | --- | --- | --- | --- | --- | --- | --- | --- | --- |
| Outcome Variable | Bivariate^1^ | |  | Partial^2^ | |  | Bivariate^1^ | |  | Partial^2^ | |
|  | *r* | *p* |  | *r* | *p* |  | *r* | *p* |  | *r* | *p* |
| *Striking Abilities* |  |  |  |  |  |  |  |  |  |  |  |
| Striking accuracy | **.14** | **.002** |  | .09 | .08 |  | **.13** | **.003** |  | .07 | .13 |
| Total strikes landed | -.04 | .32 |  | .05 | .32 |  | -.03 | .55 |  | .03 | .61 |
| Total strikes attempted | -.07 | .13 |  | .02 | .70 |  | -.05 | .28 |  | .00 | .97 |
| Strikes landed per minute | .05 | .25 |  | .03 | .59 |  | .02 | .64 |  | .01 | .88 |
| Strikes landed in standing position | -.05 | .26 |  | .03 | .50 |  | -.05 | .30 |  | .00 | .99 |
| Strikes landed in clinch position | -.03 | .47 |  | .04 | .41 |  | .00 | .99 |  | .04 | .43 |
| Strikes landed in ground position | -.00 | .83 |  | .06 | .21 |  | .03 | .51 |  | .08 | .12 |
| Strikes landed to the opponent’s head | -.05 | .23 |  | .03 | .54 |  | -.03 | .52 |  | .02 | .74 |
| Strikes landed to the opponent’s body | -.03 | .46 |  | .06 | .25 |  | -.02 | .72 |  | .04 | .39 |
| Strikes landed to the opponent’s legs | -.02 | .53 |  | .05 | .31 |  | -.04 | .40 |  | .01 | .93 |
|  |  |  |  |  |  |  |  |  |  |  |  |
| *Grappling Abilities* |  |  |  |  |  |  |  |  |  |  |  |
| Grappling accuracy | .08 | .08 |  | .09 | .09 |  | .09 | .06 |  | .10 | .053 |
| Total takedowns landed | -.02 | .73 |  | .05 | .39 |  | .08 | .14 |  | **.14** | **.01** |
| Total takedowns attempted | -.06 | .23 |  | -.00 | .97 |  | .04 | .42 |  | .08 | .11 |
| Landed takedowns per 15 minutes | .03 | .57 |  | .02 | .69 |  | **.10** | **.03** |  | .09 | .07 |
| Attempted submissions per 15 minutes | -.00 | .94 |  | -.04 | .48 |  | .05 | .37 |  | .02 | .78 |
|  |  |  |  |  |  |  |  |  |  |  |  |
| *Defensive Abilities* |  |  |  |  |  |  |  |  |  |  |  |
| Striking defense | -.06 | .21 |  | .00 | .92 |  | -.07 | .11 |  | -.01 | .82 |
| Takedown defense | -.02 | .61 |  | -.06 | .25 |  | -.03 | .56 |  | -.06 | .25 |
| Losses by KO/TKO | -.05 | .20 |  | -.03 | .48 |  | -.03 | .42 |  | -.06 | .24 |
| Losses by submission | -.04 | .30 |  | -.01 | .92 |  | -.03 | .46 |  | -.02 | .75 |
| Losses by decision | **-.09** | **.04** |  | -.02 | .72 |  | **-.09** | **.04** |  | -.06 | .20 |
|  |  |  |  |  |  |  |  |  |  |  |  |
| *Fighting Wins by Type* |  |  |  |  |  |  |  |  |  |  |  |
| Wins by KO/TKO | .08 | .07 |  | .05 | .29 |  | .04 | .34 |  | -.01 | .89 |
| Wins by submission | -.06 | .13 |  | -.05 | .35 |  | .05 | .29 |  | .07 | .13 |
| Wins by decision | -.01 | .75 |  | .04 | .40 |  | .06 | .18 |  | **.11** | **.03** |

*Note.* Partial correlations include the same covariates as previous tables, and pairwise deletion was used.

**Study 3: Individual Fight Data**

**Results**

**fWHRlid (Manual)**

***Fighting Success***

Results showed no significant association between fWHRlid (manual) and focal outcome (*β* = .23 ± .18, *X^2^* = 1.29, *p* = .20), such that those with larger fWHRs were not significantly more likely to win the fight. There was also no significant interaction between fWHRlid (manual) and the method of resolution on the focal outcome (*β* = -.06 ± .10, *X^2^* = -.61, *p* = .54), such that those with larger fWHRs were not significantly more likely to win or lose via a specific strategy (i.e., via decision, submission, KO/TKO).

***Aggression***

There were no significant associations between fWHRlid (manual) and significant strikes landed (*β* = .01 ± .04, *t* = .13, 95% CI [-.08, .09], *p* = .90), significant strikes attempted (*β* = -.01 ± .04, *t* = -.22, 95% CI [-.08, .07], *p* = .83), striking accuracy (*β* = .02 ± .04, *t* = .61, 95% CI [-.05, .09], *p* = .54), takedowns landed (*β* = -.01 ± .04, *t* = -.12, 95% CI [-.09, .08], *p* .91), or takedowns attempted (*β* = -.03 ± .05, *t* = -.65, 95% CI [-.12, .06], *p* = .52). However, there was a significant association between fWHRlid and grappling accuracy (*β* = .09 ± .04, *t* = 2.09, 95% CI [.01, .17], *p* = .04), such that those with larger fWHRs were more skilled grapplers. This latter effect was performed on the simplest model with model specification via backwards elimination (6 control variables) but this effect also held in the model with all 17 control variables (*β* = .09 ± .04, *t* = 2.09, 95% CI [.01, .17], *p* = .04) but not when there were no covariates (*β* = .04 ± .03, *t* = 1.22, 95% CI [.02, .10], *p* = .23).

**fWHRbrow (Automatic)**

***Fighting Success***

Results showed no significant association between fWHRbrow (automatic) and focal outcome (*β* = -.02 ± 0.17, *X^2^* = -.09, *p* = .93), such that those with larger fWHRs were not significantly more likely to win the fight. There was also no significant interaction between fWHRbrow (automatic) and the method of resolution on the focal outcome (*β* = .05 ± .09, *X^2^* = .49, *p* = .62), such that those with larger fWHRs were not significantly more likely to win or lose via a specific strategy (i.e., via decision, submission, KO/TKO).

***Aggression***

There were no significant associations between fWHRbrow (automatic) and significant strikes landed (*β* = -.02 ± .04, *t* = .50, 95% CI [-.06, .11], *p* = .61), significant strikes attempted (*β* = .01 ± .04, *t* = .23, 95% CI [-.07, .09], *p* = .82), striking accuracy (*β* = .01 ± .04, *t* = .35, 95% CI [-.06, .08], *p* = .72), takedowns landed (*β* = .04 ± .04, *t* = .90, 95% CI [-.05, .13], *p* = .37), or takedowns attempted (*β* = .01 ± .05, *t* = .20, 95% CI [-.08, .10], *p* = .84). However, there was a significant association between fWHRbrow (automatic) and grappling accuracy (*β* = .11 ± .04, *t* = 2.61, 95% CI [.03, .19], p = .01), such that those with larger fWHRs were more skilled grapplers. This latter effect was performed on the simplest model with model specification via backwards elimination (6 control variables) but this effect also held in the model with all 17 control variables (*β* = .11 ± .04, *t* = 2.68, 95% CI [.03, .19], p = .01) and when there were no covariates (*β* = .09 ± .03, *t* = 2.97, 95% CI [.03, .15], p = .003).

**fWHRlid (Automatic)**

***Fighting Success***

Results showed no significant association between fWHRlid (automatic) and focal outcome (*β* = .08 ± 0.17, *X^2^* = .48, *p* = .63), such that those with larger fWHRs were not significantly more likely to win the fight. There was also no significant interaction between fWHRlid (automatic) and the method of resolution on the focal outcome (*β* < .001 ± .09, *X^2^* = .002, *p* = .99), such that those with larger fWHRs were not significantly more likely to win or lose via a specific strategy (i.e., via decision, submission, KO/TKO).

***Aggression***

There were no significant associations between fWHRlid (automatic) and significant strikes landed (*β* = .04 ± .04, *t* = .95, 95% CI [-.04, .12], *p* = .34), significant strikes attempted (*β* = .03 ± .04, *t* = .67, 95% CI [-.05, .10], *p* = .50), striking accuracy (*β* = .01 ± .04, *t* = -.14, 95% CI [-.06, .07]), *p* = .89, takedowns landed (*β* = .03 ± .04, *t* = .70, 95% CI [-.05, .11], *p* = .49), or takedowns attempted (*β* = .01 ± .04, *t* = -.11, 95% CI [-.09, .08], *p* = .91). However, there was a significant association between fWHRlid (automatic) and grappling accuracy (*β* = .09 ± .04, *t* = 2.27, 95% CI [.01, .17], *p* = .02), such that those with larger fWHRs were more skilled grapplers. This latter effect was performed on the simplest model with model specification via backwards elimination (6 control variables) but this effect also held in the model with all 17 control variables (*β* = .09 ± .04, *t* = 2.36, 95% CI [.01, .17], p = .02) and when there were no covariates (*β* = .07 ± .04, *t* = 2.32, 95% CI [.01, .13], p = .02).

**Random Slopes for Focal and Non-Focal fWHR**

The focal and non-focal fighters’ fWHR were modelled into the random slopes, but frequently resulted in a singular fit or a convergence error, making the parameters less interpretable. While effect sizes and p-values could be computed and are therefore reported, confidence intervals could not be reliably computed and are therefore not reported; nonetheless, results reported here are largely similar to the intercepts-only models reported earlier, which should be given credence because they did not result in a singular fit or convergence error. Interested readers are directed to the publicly available R code and datasets located on the OSF.

**fWHRlid (Manual)**

***Fighting Success***

Results showed no significant association between fWHRlid (manual) and focal outcome (*β* = .23 ± .18, *X^2^* = 1.28, *p* = .20), such that those with larger fWHRs were not significantly more likely to win the fight. There was also no significant interaction between fWHRlid (manual) and the method of resolution on the focal outcome (*β* = -.06 ± .10, *X^2^* = -.59, *p* = .56), such that those with larger fWHRs were not significantly more likely to win or lose via a specific strategy (i.e., via decision, submission, KO/TKO).

***Aggression***

There were no significant associations between fWHRlid (manual) and significant strikes landed (*β* = .02 ± .04, *t* = .42, *p* = .67), significant strikes attempted (*β* = -.003 ± .04, *t* = -.08, *p* = .93), striking accuracy (*β* = .03 ± .04, *t* = .74, *p* = .46), takedowns landed (*β* = -.02 ± .05, *t* = -.32, *p* = .75), or takedowns attempted (*β* = -.03 ± .05, *t* = -.57, *p* = .57). However, there was a close-to-significant association between fWHRlid and grappling accuracy (*β* = .08 ± .04, *t* = 1.96, *p* = .051), such that those with larger fWHRs were more skilled grapplers. This latter effect was performed on the simplest model with model specification via backwards elimination (6 control variables) but this same effect held in the model with all 17 control variables (*β* = .08 ± .04, *t* = 1.98, *p* = .049). There was a close-to-significant association between fWHRlid and grappling accuracy when there were no control variables (*β* = .07 ± .04, *t* = 1.83, *p* = .068).

**fWHRbrow (Manual)**

***Fighting Success***

Results showed no significant association between fWHRbrow (manual) and focal outcome (*β* = .08 ± .15, *X^2^* = -0.56, *p* = .58), such that those with larger fWHRs were not significantly more likely to win the fight. There was also no significant interaction between fWHRbrow (manual) and the method of resolution on the focal outcome (*β* = -.10 ± .09, *X^2^* = 1.21, *p* = .23), such that those with larger fWHRs were not significantly more likely to win or lose via a specific strategy (i.e., via decision, submission, KO/TKO).

***Aggression***

There were no significant associations between fWHRbrow (manual) and significant strikes landed (*β* = .02 ± .04, *t* = -.39, *p* = .70), significant strikes attempted (*β* = -.03 ± .04, *t* = -.70, *p* = .48), striking accuracy (*β* = .04 ± .04, *t* = 1.17, *p* = .25), takedowns landed (*β* = -.03 ± .04, *t* = .61, *p* = .55), or takedowns attempted (*β* = .02 ± .05, *t* = .36, *p* = .73). While there was no effect in the most complex model with 17 control variables between fWHRbrow and grappling accuracy (*β* = .004 ± .04, *t* = .08, *p* = .94), there was a significant association between fWHRbrow and grappling accuracy in the simplest model (based on AIC scores in lowest-is-best format) with 6 control variables (*β* = .12 ± .04, *t* = 3.14, *p* = .002). This association between fWHRbrow and grappling accuracy also held when there were no control variables (*β* = .10 ± .03, *t* = 3.01, *p* = .003).

**fWHRbrow (Automatic)**

***Fighting Success***

Results showed no significant association between fWHRbrow (automatic) and focal outcome (*β* = -.01 ± 0.17, *X^2^* = -.05, *p* = .96), such that those with larger fWHRs were not significantly more likely to win the fight. There was also no significant interaction between fWHRbrow (automatic) and the method of resolution on the focal outcome (*β* = .05 ± .09, *X^2^* = .47, *p* = .64), such that those with larger fWHRs were not significantly more likely to win or lose via a specific strategy (i.e., via decision, submission, KO/TKO).

***Aggression***

There were no significant associations between fWHRbrow (automatic) and significant strikes landed (*β* = .02 ± .04, *t* = .50, *p* = .62), significant strikes attempted (*β* = .01 ± .04, *t* = .27, *p* = .79), striking accuracy (*β* = .01 ± .04, *t* = .33, *p* = .74), takedowns landed (*β* = .05 ± .05, *t* = .99, *p* = .32), or takedowns attempted (*β* = .01 ± .05, *t* = .14, *p* = .89). However, there was a significant association between fWHRbrow (automatic) and grappling accuracy (*β* = .11 ± .04, *t* = 2.54, p = .01), such that those with larger fWHRs were more skilled grapplers. This latter effect was performed on the simplest model with model specification via backwards elimination (6 control variables) but this effect also held in the model with all 17 control variables (*β* = .11 ± .04, *t* = 2.56, p = .01). This association between fWHRbrow and grappling accuracy also held when there were no control variables (*β* = .11 ± .04, *t* = 3.12, *p* = .003).

**fWHRlid (Automatic)**

***Fighting Success***

Results showed no significant association between fWHRlid (automatic) and focal outcome (*β* = .08 ± 0.17, *X^2^* = .47, *p* = .64), such that those with larger fWHRs were not significantly more likely to win the fight. There was also no significant interaction between fWHRlid (automatic) and the method of resolution on the focal outcome (*β* = .004 ± .09, *X^2^* = .04, *p* = .97), such that those with larger fWHRs were not significantly more likely to win or lose via a specific strategy (i.e., via decision, submission, KO/TKO).

***Aggression***

There were no significant associations between fWHRlid (automatic) and significant strikes landed (*β* = .04 ± .04, *t* = .97, *p* = .33), significant strikes attempted (*β* = .03 ± .04, *t* = .66, *p* = .51), striking accuracy (*β* = .004 ± .04, *t* = .10, *p* = .92), takedowns landed (*β* = .05 ± .05, *t* = 1.02, *p* = .31), or takedowns attempted (*β* = .01 ± .05, *t* = .16, *p* = .87). However, there was a significant association between fWHRlid (automatic) and grappling accuracy (*β* = .09 ± .04, *t* = 2.23, *p* = .03), such that those with larger fWHRs were more skilled grapplers. This latter effect was performed on the simplest model with model specification via backwards elimination (6 control variables) but this effect also held in the model with all 17 control variables (*β* = .09 ± .04, *t* = 2.29, p = .03). This association between fWHRbrow and grappling accuracy also held when there were no control variables (*β* = .09 ± .04, *t* = 2.40, *p* = .02).

**Random Slopes for Focal fWHR**

Focal fighters’ fWHR was modelled into the random slopes, rather than both focal and non-focal fighters’ fWHR, but again frequently resulted in a singular fit or a convergence error, making the parameters less interpretable. As with the previous analyses, while effect sizes and p-values could be computed and are therefore reported, confidence intervals could not be reliably computed and are therefore not reported; again, all results reported here are identical to the intercepts-only models reported earlier. Interested readers are directed to the publicly available R code and datasets located on the OSF.

**fWHRlid (Manual)**

***Fighting Success***

Results showed no significant association between fWHRlid (manual) and focal outcome (*β* = .23 ± .18, *X^2^* = 1.29, *p* = .19), such that those with larger fWHRs were not significantly more likely to win the fight. There was also no significant interaction between fWHRlid (manual) and the method of resolution on the focal outcome (*β* = -.06 ± .10, *X^2^* = -.61, *p* = .54), such that those with larger fWHRs were not significantly more likely to win or lose via a specific strategy (i.e., via decision, submission, KO/TKO).

***Aggression***

There were no significant associations between fWHRlid (manual) and significant strikes landed (*β* = .01 ± .05, *t* = .26, *p* = .79), significant strikes attempted (*β* = -.01 ± .04, *t* = -.14, *p* = .89), striking accuracy (*β* = .03 ± .04, *t* = .71, *p* = .48), takedowns landed (*β* = -.01 ± .05, *t* = -.22, *p* = .82), or takedowns attempted (*β* = -.03 ± .05, *t* = -.56, *p* = .58). There was a significant association between fWHRlid and grappling accuracy (*β* = .09 ± .04, *t* = 2.15, *p* = .03), such that those with larger fWHRs were more skilled grapplers. This latter effect was performed on the simplest model with model specification via backwards elimination (6 control variables) and this same effect held in the model with all 17 control variables (*β* = .09 ± .04, *t* = 2.14, *p* = .03). This association between fWHRlid and grappling accuracy was not significant when there were no control variables (*β* = .04 ± .03, *t* = 1.21, *p* = .23).

**fWHRbrow (Manual)**

***Fighting Success***

Results showed no significant association between fWHRbrow (manual) and focal outcome (*β* = .09 ± .15, *X^2^* = -0.56, *p* = .57), such that those with larger fWHRs were not significantly more likely to win the fight. There was also no significant interaction between fWHRbrow (manual) and the method of resolution on the focal outcome (*β* = -.10 ± .09, *X^2^* = 1.22, *p* = .22), such that those with larger fWHRs were not significantly more likely to win or lose via a specific strategy (i.e., via decision, submission, KO/TKO).

***Aggression***

There were no significant associations between fWHRbrow (manual) and significant strikes landed (*β* = .02 ± .04, *t* = -.39, *p* = .70), significant strikes attempted (*β* = -.03 ± .04, *t* = -.74, *p* = .46), striking accuracy (*β* = .04 ± .04, *t* = 1.18, *p* = .24), takedowns landed (*β* = -.03 ± .04, *t* = .71, *p* = .48), or takedowns attempted (*β* = .02 ± .05, *t* = .36, *p* = .73). While there was no effect in the most complex model with 17 control variables between fWHRbrow and grappling accuracy (*β* = .003 ± .04, *t* = .07, *p* = .95), there was a significant association between fWHRbrow and grappling accuracy in the simplest model (based on AIC scores in lowest-is-best format) with 6 control variables (*β* = .12 ± .04, *t* = 3.21, *p* = .001). This association between fWHRbrow and grappling accuracy also held when there were no control variables (*β* = .07 ± .03, *t* = 2.56, *p* = .01).

**fWHRbrow (Automatic)**

***Fighting Success***

Results showed no significant association between fWHRbrow (automatic) and focal outcome (*β* = -.02 ± .17, *X^2^* = -.09, *p* = .93), such that those with larger fWHRs were not significantly more likely to win the fight. There was also no significant interaction between fWHRbrow (automatic) and the method of resolution on the focal outcome (*β* = .05 ± .09, *X^2^* = .50, *p* = .62), such that those with larger fWHRs were not significantly more likely to win or lose via a specific strategy (i.e., via decision, submission, KO/TKO).

***Aggression***

There were no significant associations between fWHRbrow (automatic) and significant strikes landed (*β* = .02 ± .04, *t* = .50, *p* = .62), significant strikes attempted (*β* = .01 ± .04, *t* = .25, *p* = .81), striking accuracy (*β* = .01 ± .04, *t* = .40, *p* = .69), takedowns landed (*β* = .05 ± .05, *t* = .94, *p* = .35), or takedowns attempted (*β* = .01 ± .05, *t* = .13, *p* = .90). However, there was a significant association between fWHRbrow (automatic) and grappling accuracy (*β* = .11 ± .04, *t* = 2.60, p = .01), such that those with larger fWHRs were more skilled grapplers. This latter effect was performed on the simplest model with model specification via backwards elimination (6 control variables) but this effect also held in the model with all 17 control variables (*β* = .11 ± .04, *t* = 2.62, p = .01). This association between fWHRbrow and grappling accuracy also held when there were no control variables (*β* = .10 ± .03, *t* = 3.09, *p* = .003).

**fWHRlid (Automatic)**

***Fighting Success***

Results showed no significant association between fWHRlid (automatic) and focal outcome (*β* = .08 ± 0.17, *X^2^* = .47, *p* = .63), such that those with larger fWHRs were not significantly more likely to win the fight. There was also no significant interaction between fWHRlid (automatic) and the method of resolution on the focal outcome (*β* = .0001 ± .09, *X^2^* = .001, *p* = .99), such that those with larger fWHRs were not significantly more likely to win or lose via a specific strategy (i.e., via decision, submission, KO/TKO).

***Aggression***

There were no significant associations between fWHRlid (automatic) and significant strikes landed (*β* = .04 ± .04, *t* = .97, *p* = .33), significant strikes attempted (*β* = .03 ± .04, *t* = .66, *p* = .51), striking accuracy (*β* = .01 ± .04, *t* = .37, *p* = .71), takedowns landed (*β* = .04 ± .05, *t* = .94, *p* = .35), or takedowns attempted (*β* = .01 ± .05, *t* = .16, *p* = .87). However, there was a significant association between fWHRlid (automatic) and grappling accuracy (*β* = .09 ± .04, *t* = 2.34, *p* = .02), such that those with larger fWHRs were more skilled grapplers. This latter effect was performed on the simplest model with model specification via backwards elimination (6 control variables) but this effect also held in the model with all 17 control variables (*β* = .09 ± .04, *t* = 2.31, p = .02). This association between fWHRlid and grappling accuracy also held when there were no control variables (*β* = .08 ± .03, *t* = 2.44, *p* = .02).
